# Transcript Expression Analysis of Putative *Trypanosoma brucei* GPI-Anchored Surface Proteins during Development in the Tsetse and Mammalian Hosts

**DOI:** 10.1371/journal.pntd.0001708

**Published:** 2012-06-19

**Authors:** Amy F. Savage, Gustavo C. Cerqueira, Sandesh Regmi, Yineng Wu, Najib M. El Sayed, Serap Aksoy

**Affiliations:** 1 Division of Epidemiology of Microbial Diseases, Yale School of Public Health, Yale University, New Haven, Connecticut, United States of America; 2 Department of Cell Biology and Molecular Genetics, Maryland Pathogen Research Institute (MPRI), University of Maryland, College Park, Maryland, United States of America; 3 Center for Bioinformatics and Computational Biology, College of Chemical & Life Sciences, University of Maryland, College Park, Maryland, United States of America; National Institute of Allergy and Infectious Diseases, United States of America

## Abstract

Human African Trypanosomiasis is a devastating disease caused by the parasite *Trypanosoma brucei*. Trypanosomes live extracellularly in both the tsetse fly and the mammal. Trypanosome surface proteins can directly interact with the host environment, allowing parasites to effectively establish and maintain infections. Glycosylphosphatidylinositol (GPI) anchoring is a common posttranslational modification associated with eukaryotic surface proteins. In *T. brucei*, three GPI-anchored major surface proteins have been identified: variant surface glycoproteins (VSGs), procyclic acidic repetitive protein (PARP or procyclins), and *brucei* alanine rich proteins (BARP). The objective of this study was to select genes encoding predicted GPI-anchored proteins with unknown function(s) from the *T. brucei* genome and characterize the expression profile of a subset during cyclical development in the tsetse and mammalian hosts. An initial *in silico* screen of putative *T. brucei* proteins by Big PI algorithm identified 163 predicted GPI-anchored proteins, 106 of which had no known functions. Application of a second GPI-anchor prediction algorithm (FragAnchor), signal peptide and trans-membrane domain prediction software resulted in the identification of 25 putative hypothetical proteins. Eighty-one gene products with hypothetical functions were analyzed for stage-regulated expression using semi-quantitative RT-PCR. The expression of most of these genes were found to be upregulated in trypanosomes infecting tsetse salivary gland and proventriculus tissues, and 38% were specifically expressed only by parasites infecting salivary gland tissues. Transcripts for all of the genes specifically expressed in salivary glands were also detected in mammalian infective metacyclic trypomastigotes, suggesting a possible role for these putative proteins in invasion and/or establishment processes in the mammalian host. These results represent the first large-scale report of the differential expression of unknown genes encoding predicted *T. brucei* surface proteins during the complete developmental cycle. This knowledge may form the foundation for the development of future novel transmission blocking strategies against metacyclic parasites.

## Author Summary

Human African Trypanosomiasis (HAT) is a fatal disease caused by African trypanosomes and transmitted by an infected tsetse fly. Presently, there are no vaccines to prevent mammalian infections. Proteins expressed on the trypanosome surface can influence the host environment and allow for their transmission. Potentially accessible to the adaptive immune systems of vertebrate hosts, these proteins could serve as future vaccine targets. Identification and characterization of these currently unknown proteins can help us develop strategies to alter the host environment, making it inhospitable for the parasite, thereby reducing disease transmission. While there is extensive knowledge about trypanosome development in the mammalian host, less is known about the molecular events in the tsetse fly, particularly the salivary gland stages. We used an *in silico* approach to identify putative surface proteins from the known genome sequence of *Trypanosoma brucei*, and we describe the stage specific expression of these genes during development in the tsetse fly and mammalian host. Our findings show that a majority of unknown transcripts encoding predicted surface proteins are expressed by the parasites infecting tsetse salivary glands. These data will help focus future investigations into transmission-blocking approaches targeting the expressed antigens of trypanosomes infecting tsetse salivary glands.

## Introduction

Sleeping Sickness, or Human African Trypanosomiasis (HAT), is a fatal parasitic disease transmitted by the bite of an infected tsetse (*Glossina* spp.) fly. The disease agents are the extracellular protozoan parasites belonging to the *Trypanosoma brucei* species complex. It is estimated that 60 million people in 36 African nations are at risk for HAT. The same parasite species complex also infects animals causing nagana, an economically important disease of livestock in Africa. There are no mammalian vaccines for disease control and the drugs used for chemotherapy have major adverse effects, are difficult to administer and have decreased efficacy in light of the emergence of parasite drug resistance. A number of disease control strategies, mainly focused on vector control and treatment of infections, have been applied. These are often successful in the short term, although a sustainable long-term solution remains unidentified.

African trypanosomes undergo multiple differentiation steps as they complete their life cycle in the challenging environments of the mammalian and invertebrate hosts. Trypanosomes circulating in the mammalian bloodstream (bloodstream form, BSF) are found as either long slender forms that perpetuate the infection in the mammal, or as short stumpy forms that are infective to the tsetse fly. In the mammalian host, BSF parasites evade the adaptive immune system by changing their surface coat molecules in a process known as antigenic variation [1]. Antigenic variation has effectively prevented the development of mammalian vaccines to date.

In the tsetse flies, a strong immune response apparently clears the parasites in the majority (over 95%) of challenged tsetse [2] but those parasitized flies remain infected for their lifetime and contribute to disease transmission. Once acquired in an infected bloodmeal, trypanosomes undergo several stages of differentiation in the fly before they are transmissible to the mammalian host [3]. In the midgut, the stumpy BSF parasites differentiate to the procyclic form (PF) parasites. Although the majority of flies can clear parasite infections at this stage [2], in flies where the PF cells survive, trypanosomes migrate anteriorly to the proventriculus, and differentiate initially into the mesocyclic trypomastigote, then long and short epimastigotes. It is thought that only the short epimastigotes can invade the salivary glands, where they attach and differentiate ultimately giving rise to the free mammalian infective metacyclic trypomastigotes (MCF), which are transferred to the mammalian host in saliva as the infected fly blood feeds. Only the BSF and PF developmental stages of *T. brucei* can be maintained in culture *in vitro*. The remaining developmental stages of the parasite can only be maintained in tsetse, making the access to and evaluation of these life stages difficult.

The genomes of several related kinetoplastid parasites have been published, including *T. brucei brucei* and *Trypanosoma brucei gambiense* [4]–[8]. Improved technologies such as RNA sequencing have identified over 1,000 new transcripts in *T. b. brucei* [9]. Particularly relevant for disease control tools are surface expressed proteins that interact with the host environment, and specifically with the host immune system. Protein features that are suggestive of surface expression are associated signal peptides, trans-membrane domains, and glycosylphosphatidylinositol (GPI) anchor attachment domains. Many GPI-anchored proteins in mammalian systems have been shown or predicted to have hydrolytic activity, or serve as receptors or adhesion molecules, while some are suggested to be involved in trans-membrane signaling or membrane trafficking [10], [11].

The two well-studied GPI-anchored surface coat proteins of *T. brucei* are the variant surface glycoproteins (VSGs) and procyclins, expressed by the BSF and PF cells, respectively. The VSG coat of the BSF trypanosome allows the parasite to evade the adaptive immune response and therefore persist in the mammalian host. The procyclins were initially thought to shield PF parasites from the digestive enzymes of the fly midgut [12], but procyclin-null mutant trypanosomes were subsequently found to be capable of infecting tsetse [13]. BARP, a third GPI-anchored surface protein family identified in *T. brucei* [14] is expressed by immature salivary gland stages [15]. Functional assessment of the BARP proteins have not yet been described, so it is unknown if trypanosome survival or maturation in the salivary gland environment would be influenced in their absence. The serum resistance associated protein (SRA), which allows the survival of *Trypanosoma brucei rhodesiense* in the human host was recently determined to be GPI-anchored [16], demonstrating the role of GPI-anchored proteins in the host-range of this pathogen. Additionally, a sub-unit of the transferrin receptor (ESAG6) was also demonstrated to be GPI-anchored [17], and work continues on the characterization of this molecule.

Here, we report on the differential expression of transcripts corresponding to putative GPI anchored proteins with unknown functions in *T. brucei*. The selected genes were initially identified *in silico* using the Big PI and subsequently by the FragAnchor GPI-prediction algorithms. The signal peptide and trans-membrane domains of the putative proteins were also analyzed *in silico*. Gene expression data was obtained from parasites infecting the tsetse and mammalian hosts. We discuss the implications of the observed transcript expression profiles with regard to parasite survival and transmission processes, with consideration of the mammalian infective metacyclic trypomastigote.

## Materials and Methods

### Ethics statement

This experiment was carried out in strict accordance with the recommendations in the Office of Laboratory Animal Welfare at the National Institutes of Health and the Yale University Institutional Animal Care and Use Committee. The experimental protocol was reviewed and approved by the Yale University Institutional Animal Care and Use Committee (Protocol 2011-07266).

### *In silico* analysis

Genes encoding putative GPI anchor attachment domains were identified *in silico* and manually curated during the annotation of the first publicly available *T. brucei brucei* strain 927 genome sequence [4]. GPI anchor predictions were made by the consortium using the publicly available software Big-PI Predictor (http://mendel.imp.ac.at/gpi/gpi_server.html) [18]. The known GPI anchored protein families, such as VSG and procyclin, were removed from the resulting list. A second program, FragAnchor (http://navet.ics.hawaii.edu/~fraganchor/NNHMM/NNHMM.html), was applied to genome annotation data from version 4 of the *T. brucei* genome [19]. Non-VSG, non-procyclin genes were categorized as hypothetical, hypothetical conserved, or annotated with known functions, according to the parameters set by the *T. brucei* genome consortium. Interpro domains associated to these genes were retrieved from TriTrypDB (http://tritrypdb.org/tritrypdb/). Gene products with less than 36% identity to a match in the public databases were considered hypothetical proteins, having no known function. When protein identity levels of 36% and higher to other hypothetical proteins were detected, the protein was considered hypothetical conserved. Hypothetical conserved genes, which were predicted to have homologs within the *T. brucei* genome, were further classified as hypothetical gene family members. Homology to other kinetoplastid species was determined using either existing data on the Sanger *T. brucei* website (for *Leishmania major* and *Trypanosoma cruzi*, (TriTryp)), or by using the omniBLAST protein search function on the Sanger GeneDB website (http://www.genedb.org). Signal peptide and cleavage site predictions were determined by SignalP (http://www.cbs.dtu.dk/services/SignalP/) [20]. Trans-membrane predictions were made using DAS Software (http://www.sbc.su.se/~miklos/DAS/) [21]. Predictions of glycosylation sites were performed using the NetNGlyc 1.0 (http://www.cbs.dtu.dk/services/NetNGlyc/) and NetOGlyc Servers (http://www.cbs.dtu.dk/services/NetOGlyc/) [22]. All prediction software is publically available on the internet.

### Parasite strains and tsetse infections

The parasite strains used were *T. b. brucei* RUMP 503 and *T. b. rhodesiense* YTAT 1.1. For gene expression analysis, RNA was prepared from BSF *T. b. rhodesiense* expanded in rats. Trypanosomes were harvested from infected blood at peak parasitemia using DEAE cellulose chromatography [23], [24]. For fly infections, BSF *T. b. brucei* expanded in rats were cryopreserved for subsequent use. Newly emerged male flies from the *Glossina morsitans morsitans* colony maintained in the Yale insectary received 2×10^6^–2×10^7^/mL *T. b. brucei* parasites in defibrinated bovine blood meal diet using an artificial membrane system [25]. After a single parasite challenge, flies were maintained on defibrinated bovine blood provided every other day.

### Tissue dissections, RNA isolation and cDNA generation

Flies were dissected after a minimum of 40 days post infection (dpi) and 72 hrs after their last blood meal. Salivary gland (SG) infection status was microscopically determined on a Zeiss Axiostar Plus light microscope at 400×. Infected SG, proventriculus (PV) and midgut (MG) tissues from the same flies were collected in Trizol, vortexed, and midguts were homogenized immediately. Metacyclic form (MCF) parasites were obtained by collecting the blood remaining on the feeding apparatus after flies with mature SG infections were fed. Blood was collected in PSG buffer (0.04 M Na_2_HPO_4_ 2 H_2_0, 0.006 M NaH_2_PO_4_ 2 H_2_O, 0.07 M NaCl, to pH 8.0 with 1 M H_2_PO_4_), centrifuged 5 min. at 3000 rpm, and the pellet was resuspended in Trizol and stored at −20°C until RNA isolation. Total RNA was isolated from fly tissues and infected blood using Trizol extraction, according to manufacturer's instructions (catalog no. 15596-026, Invitrogen, California). Genomic DNA was removed by incubation with DNAse I, according to manufacturer's protocol (catalog no. 04716728001, Roche, Indiana). Reverse transcription was performed according to manufacturers instructions for oligo d(T) primed reactions (SuperScript II Reverse Transcriptase, catalog no. 18064-014; RNaseOUT, catalog no. 10777-019, Invitrogen, California).

### Primer design and PCR amplification

Nucleotide sequences for all experimental genes were obtained from the publicly available genome reference at the Sanger Institute (http://www.genedb.org/Homepage/Tbruceibrucei927). Primer sequences were identified by using the OligoPerfect™ Designer primer design tool (http://tools.invitrogen.com/content.cfm?pageid=9716) (see Table S1). All primer sets were used in a PCR amplification reaction with gDNA to confirm that they amplified a single gene fragment of the expected size. PCR amplification conditions were: 2 minutes hot start at 95°C, 32 cycles at (95°C for 45 s, 53°C for 45 s, 74°C for 1 min) and 74°C for 6 min. Primers used to amplify *procyclin* transcripts were designed to recognize both *EP* and *GPEET procyclin*.

### Semi-quantitative RT-PCR

The trypanosome structural gene *alpha-tubulin* was used for normalization of experimental cDNAs: trypanosome infected tsetse SG, PV, and MG, as well as BSF obtained from infected rats. Five and ten-fold serial dilutions of each cDNA pool were analyzed by PCR for the presence of *alpha-tubulin* transcripts. Cycling conditions were: 2 min at 95°C, 28 cycles at (95°C for 45 s, 53°C for 45 s, 74°C for 1 min) and 74°C for 6 min. The PCR amplification products from the different cDNA dilutions were resolved on a 1% agarose gel, visualized on a KODAK Image Station 2000R and gel images were captured using the IS2000R Image Aquire Software (Eastman Kodak Co, Rochester New York). The cDNA dilutions that resulted in PCR products of equal intensity from the different tissue samples were identified and all subsequent PCR reactions were performed using these cDNA template dilutions.

All experimental reactions were performed using the cDNA templates prepared as described above at 32 and 36 cycles for each sample in duplicate. As controls, *alpha-tubulin* and *BARP* sequences were amplified at 32 and 36 cycle reactions, respectively. Primer sequences can be found in Table S1. All amplification products were analyzed by electrophoresis and imaging as described above. Genes that resulted in no amplification products or that yielded multiple bands after amplification were excluded from further analysis (Table S2). Expression analysis was repeated for genes that yielded a product in only one tissue cDNA or for genes with unclear results due to low levels of expression.

### Quantifying gene expression levels

Gel images obtained from the 36 cycle reactions were used to obtain a semi-quantitative measurement of expression variation between different developmental samples. The values were normalized to the trypanosome *alpha-tubulin* control to account for variation between the four experimental tissue samples (SG, PV, MG, and BSF) and experimental runs. The adjusted expression values based on *alpha-tubulin* levels were used to categorize the expression profile of experimental genes. Based on these adjusted values, the fold change was calculated for the four developmental samples tested, for each gene yielding expression data. Where no expression could be detected, that transcript was classified as not detected (nd). If expression in one tissue was at least 2-fold higher than any other tissue, that gene was classified as being specific to that tissue. Parasite gene expression was classified as preferential for a tissue (or tissues) when gene expression was detected but the levels were less than 2 fold higher than that detected in other tissues. Genes with expression levels too low to be confidently categorized, or with expression profiles not corresponding to any other category were classified as miscellaneous. Expression levels were classified as high, medium or low based on the adjusted net intensities of the most prominent band for the experimental gene. Net intensity values ≥501 were classified as high, 101–500 as medium, and 0–100 as low. All expression data are being submitted to TriTrypdb.org.

### Quantitative RT-PCR

To validate the expression profiles observed with semi-quantitative analysis, 5 genes were selected for quantitative RT-PCR (qRT-PCR). Standard curves were developed for each gene using serial dilutions of plasmids containing cloned inserts. Each standard was used to calculate transcript numbers in the experimental cDNAs tested. qRT-PCR primers and cycling conditions are listed in Table S3. All reactions were performed on an icycler iQ real time RT-PCR detection system (Bio-Rad). Three independent biological replicates of infected SG, PV and MG tissues were used, with 2 technical replicates per sample. For comparison to the quantitative data, the semi-quantitative fold change data was evaluated based on the SG, PV, and MG data points. As no BSF samples were evaluated by qRT-PCR, the semi-quantitative data for the BSF parasites was excluded from this comparison. *Alpha-tubulin* levels were used for expression normalization. Values are represented as the mean fold change (±SEM) and statistical significance was determined using a Student's t test implemented in Microsoft Excel software.

## Results

The goal of this work was to determine the developmental and host tissue specific expression profiles of transcripts corresponding to previously undiscovered putative GPI-anchored proteins in *T. brucei*. We describe the selection of the genes encoding such predicted GPI-anchored proteins from the available *T. b. brucei* genome sequence, and their differential expression during development in tsetse tissues and the mammalian host as determined by semi-quantitative analysis. Further, quantitative expression analysis on a random subset of these genes validated the semi-quantitative results.

### *In silico* screen

An *in silico* analysis of the *T. b. brucei* strain 927 genome data using the BigPI GPI-anchor prediction software identified 163 putative proteins with GPI anchor attachment motifs. Fifty-seven of these gene products had known or predicted functions such as *BARP*, *GP63*, *trans-sialidase* and the *procyclin-associated* genes, and were excluded from further analysis (Table S4). The remaining 106 putative proteins were evaluated for the presence of conserved domains (Table S5). These putative products were further searched for glycosylation, signal peptide, and trans-membrane domains, and a second predictive algorithm for GPI-anchor attachment domain (FragAnchor) was applied (Table S6). Typical GPI anchored proteins are expected to have a signal peptide and no trans-membrane domains [26]. Our analysis reduced the initial 106 genes down to 25 genes, which were predicted to encode products with GPI-anchor attachment motifs (Table 1).

**Table 1:** Genes identified by *in silico* analysis of the *T. brucei* genome for predicted GPI-anchored proteins.

Tb ORF	Kinetoplastid conservation*	GPI anchor prediction		Signal Peptide#	Gly. Status§
		BigPI	FragAnchor		
**Hypothetical Conserved**					
*Tb927.3.2400*	TriTryp	x	x	x	
*Tb927.4.1110*	TriTryp	x	x	x	N-gly
*Tb927.4.3290*	Tco	x	x	x	
*Tb927.8.1250*	TriTryp	x	x	x	N-gly
*Tb09.211.2750*	TriTryp	x	x	x	
*Tb09.211.4155*	TriTryp	x	x	x	
*Tb927.10.990*	Tco	x	x	x	N-gly
**Gene Families**					
*Tb927.6.1310*		x	x	x	N-gly
*Tb927.7.360*		x	x	x	N-gly
*Tb927.7.380*		x	x	x	N-gly
*Tb927.7.400*		x	x	x	N-gly
*Tb927.7.420*		x	x	x	N-gly
*Tb927.7.440*	Tbg	x	x	x	N-gly
*Tb927.8.930*	Tbg, Tv	x	x	x	N-gly
*Tb927.8.950*	Tbg, Tv	x	x	x	N-gly
*Tb09.v1.0450*		x	x	x	N-gly
*Tb09.v1.0470*		x	x	x	N-gly
*Tb09.v1.0500*		x	x	x	
*Tb09.v1.0530*		x	x	x	
*Tb09.211.0010*	Tbg	x	x	x	N-gly
*Tb927.10.4390*	Tbg	x	x	x	N- & O-gly
*Tb927.10.4380*	Tbg	x	x	x	N- & O-gly
*Tb927.10.5700*	Tbg	x	x	x	N-gly
*Tb927.10.5710*	Tbg	x	x	x	N-gly
**Hypothetical**					
*Tb09.142.0410*		x	x	x	O-gly

*: TriTryp (*T. brucei*, *T. cruzi*, *L. major*); Tbg = *T. brucei gambiense*; Tco = *T. congolense*; Tv = *T. vivax*.

# signal peptide and signal sequence cleavage prediction made by publically available software.

**§:** N- or O-glycosylation status predicted by publically available software.

Conservation within the sequenced kinetoplastid genomes in addition to *T. brucei* and predicted features of the predicted protein are shown.

Of the 25 highly probable GPI-anchored gene products with unknown functions, only *Tb09.142.0410* was considered to be hypothetical, having no identified homologs. Two genes (*Tb927.4.3290* and *Tb927.10.990*) were shared only with *Trypanosoma congolense*, while five others were conserved at the level of the TriTryp genomes. Seventeen genes were identified to be members of larger gene families. Interestingly, these were not widely shared between related kinetoplastids. Only one family (*Tb927.8.930* and *Tb927.8.950*) had homologs outside of the *T. brucei* complex, and these were found in *Trypanosoma vivax*. The remaining gene family members were either detected only as repeated genes in the genome of *T. b. brucei* (9), or as having homologs in the genome of *T. b. gambiense* (6).

#### Developmental stage-regulated gene expression assay

Semi-quantitative RT-PCR analysis was used to evaluate the tissue and developmental stage-regulated expression of 81 genes, predicted to contain GPI-anchor attachment signals based on Big PI analysis. The expression profile data were validated for 5 randomly selected genes using qRT-PCR analysis.

**Figure 1:**
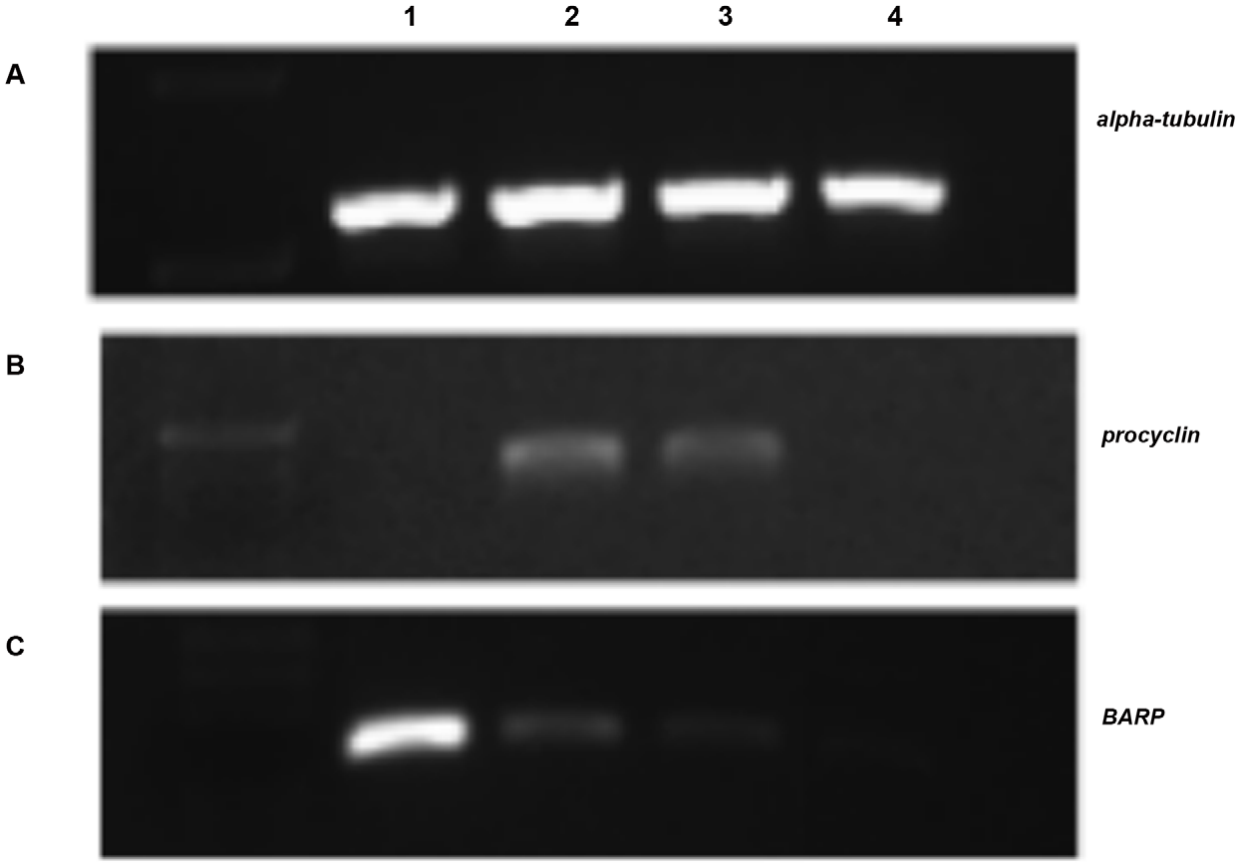
Expression profiling of three known genes from experimental cDNA templates. As experimental controls *alpha-tubulin*, *procyclin* and *BARP* gene expression was analyzed A) *alpha-tubulin*, B) *procyclin*, C) *BARP* PCR amplified from infected tsetse salivary glands (lane 1), proventriculus (lane 2), midguts (lane 3), and bloodstream form (lane 4) cDNAs.

All transcripts were analyzed by PCR amplification using normalized cDNA templates prepared from the MG, PV and SG tissues microscopically confirmed to be infected with *T. b. brucei*, and from BSF parasites obtained from infected rat blood. The normalization process is described in the methods, and an example is shown in Figure 1a. As controls, the cDNAs were also analyzed for the presence of transcripts corresponding to two known stage-regulated gene families, *procyclin* and *BARP*. Procyclin proteins are expressed by trypanosomes infecting the tsetse MG and PV. Accordingly, *procyclin* transcripts were detected only in cDNAs from these tissues (Figure 1b). *BARP* has been shown to be expressed only on the attached forms of trypanosomes in the SG [4] and PCR amplification of experimental cDNAs similarly demonstrated SG specific expression of this gene (Figure 1c).

**Figure 2:**
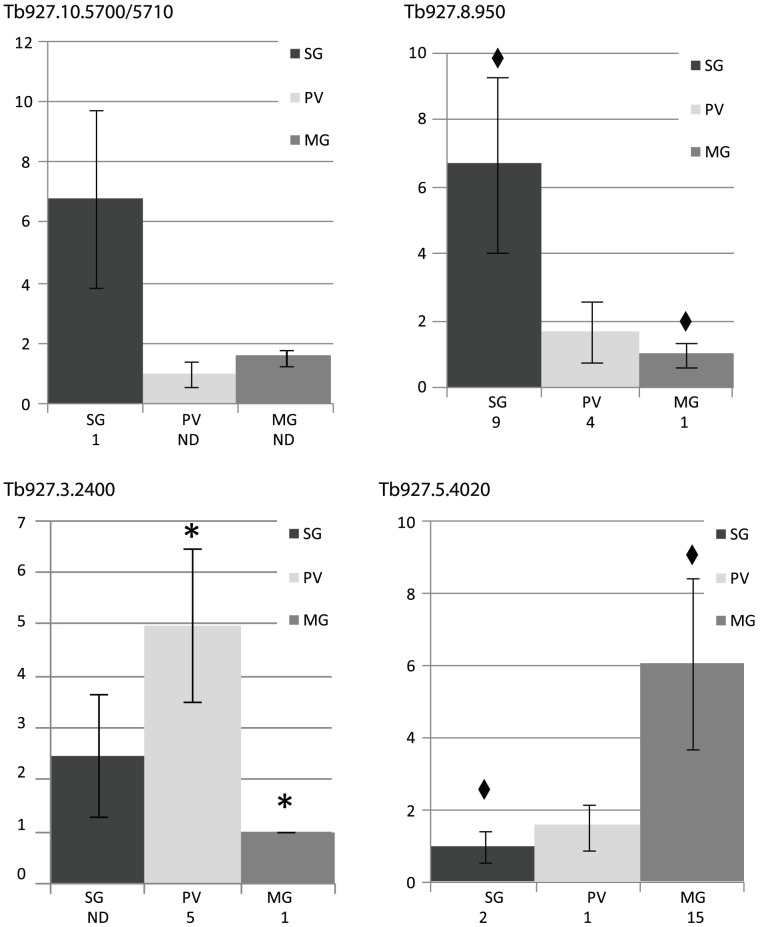
Validation of semi-quantitative RT-PCR analysis. Fold-change expression was measured by qRT-PCR analysis for randomly selected trypanosome genes in tsetse SG, PV, and MG tissues, relative to *alpha-tubulin* expression (bar graph). The values obtained by the semi-quantitative fold-change analysis for the same genes are shown as numerical data below each graph. Tb927.10.5700/5710 are two related genes amplified by the same primers. Asterisks (*) denote statistical significance (p≤0.05), diamonds (♦) denote non-significant trend p≤0.10.

The 5 genes which were randomly selected for qRT-PCR validation included SG specific, PV preferential, MG specific and miscellaneous genes, representing the high (1), medium (1), and low (3) expression level categories. The qRT-PCR data for each of these genes corresponded well to the semi-quantitative data (Figure 2). Expression of the SG specific gene *Tb927.8.950*, was at least 2-fold higher in the SG stages than in either the PV or MG stages by qRT-PCR, corresponding well to the semi-quantitative analysis. The quantitative and semi-quantitative data for the single PV preferential gene, *Tb927.3.2400*, both showed the same trend of high expression only by parasites in the PV, and the qRT-PCR results showed transcript abundance in the PV and MG samples to be significantly different. Similarly, both analyses yielded the same results for the MG specific *Tb927.5.4020*. Finally, although they were expressed at too low a level to be conclusively categorized by semi-quantitative PCR analysis, the genes *Tb927.10.5710* and *Tb927.10.5700* appeared to be SG specific based on both analyses. Large standard deviations were observed with many of the salivary gland qRT-PCR results, which are expected due to the individual variability in parasite life stage proportions in infected salivary glands. The strong correlation between the qRT-PCR and semi-quantitative RT-PCR analyses for the selected genes validated the semi-quantitative results indicating these data are representative of the *in vivo* expression profile of the evaluated unknown genes.

#### Developmental stage-regulated expression of putative GPI associated hypothetical proteins

We detected transcripts from parasitized MG, PV and SG tissues for 59 of the 81 trypanosome genes analyzed. Expression data for the 38 genes identified as “low likelihood of encoding GPI-anchored proteins” can be seen in Table S7. Expression data for the 25 genes identified as “high likelihood of encoding GPI-anchored proteins,” can be seen in Table 2. Transcripts could be detected for only 21 of the 25 genes and strikingly, most of these genes (76.2%; 16/21) were expressed by parasites infecting tsetse SG or PV tissues. If expression of a gene in stages infecting one tissue was at least 2-fold higher than expression levels measured in stages infecting another tissue, that gene was classified as being specific to stages infecting the former tissue. With that in mind, more than one-third of the analyzed genes (38.1%, 8/21) were specifically expressed by SG parasite stages (Table 2). Similarly, transcripts corresponding to 80% of the genes with “low likelihood of encoding GPI-anchored proteins” were detected in SG and PV tissues (Table S7).

**Table 2:** Normalized stage-regulated gene expression profiles and levels for predicted likely GPI anchored proteins.

Tb ORF	SG°	PV°	MG°	BSF°	Expression Level•
**SALIVARY GLAND SPECIFIC**					
*Tb927.7.360/Tb927.7.380/Tb927.7.440*	58	1	2	nd	High
*Tb927.7.400/Tb927.7.420*	34	2	1	2	High
*Tb927.6.1310*	4	nd	nd	1	High
*Tb927.8.950*	17	8	2	1	High
*Tb09.211.4155*	6	2	1	1	Medium
**SALIVARY GLAND and PROVENTRICULUS PREFERENTIAL**					
*Tb927.8.930*	4	4	1	nd	Medium
*Tb09.142.0410*	24	18	1	2	Medium
**PROVENTRICULUS PREFERENTIAL**					
*Tb927.3.2400*	nd	5	1	nd	Low
*Tb927.4.1110*	1	5	1	nd	Low
*Tb09.211.0010*	nd	1	nd	nd	Low
**PROVENTRICULUS and BSF PREFERENTIAL**					
*Tb927.10.4390*	1	7	1	4	High
*Tb09.211.2750*	3	6	1	5	Medium
*Tb09.v1.0450*	nd	1	nd	2	Low
**INSECT PREFERENTIAL**					
*Tb927.8.1250*	3	2	2	1	Medium
**CONSTITUTITIVE**					
*Tb927.10.4380*	1	2	3	1	Low
**MISCELLANEOUS**					
*Tb927.10.5710/Tb927.10.5700*	1	nd	nd	nd	Low
*Tb927.10.990*	nd	nd	1	nd	Low

**°:** SG = salivary gland, PV = proventriculus, MG = midgut, BSF = bloodstream form.

**•:** Expression level was categorized based on artificial numerical values.

nd = not detected.

Relative expression levels of experimental genes were determined by calculating relative band intensities for each PCR product relative to *alpha-tubulin* expression in the same sample. Fold change was calculated based on the value for the tissue with the lowest detectable expression for each gene.

**Figure 3:**
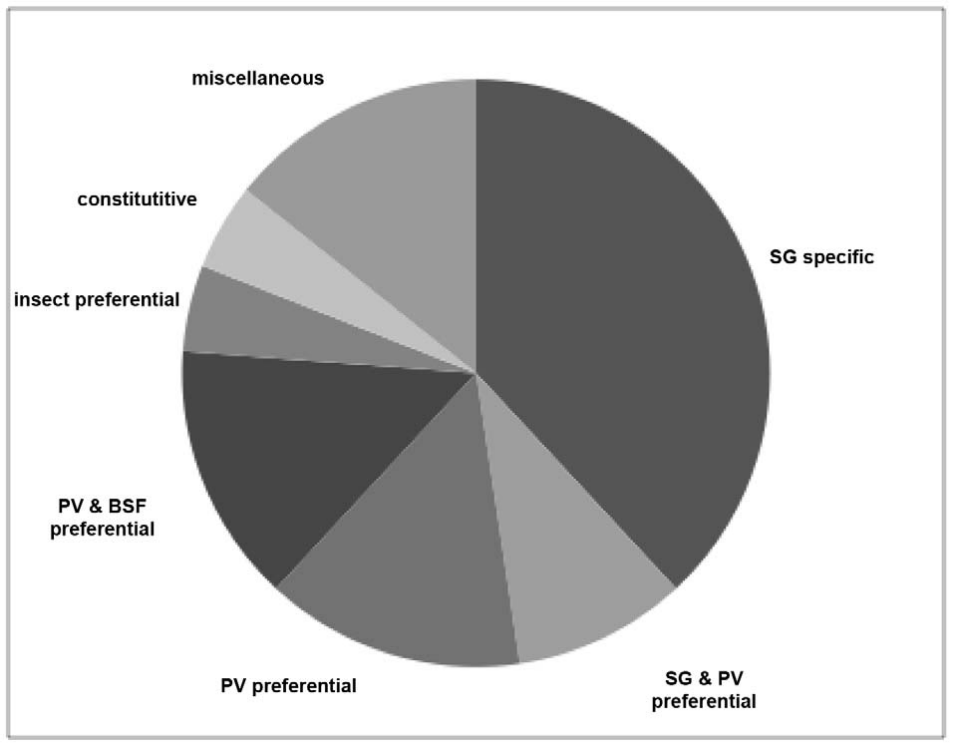
Tissue specificity of trypanosome gene expression. Percentage of trypanosome transcripts corresponding to putative probable GPI-anchored proteins detected by semi-quantitative RT-PCR by expression profile classification.

Relative gene expression levels were determined by net band intensity (Table 2). With a single exception (*Tb927.10.4390*), only SG stage-regulated trypanosome genes were highly expressed. An equal number of genes (8) were expressed at low levels, with only a small proportion of transcripts (5/21) being detected at moderate levels (Table 2). Expression profiles, which were high in a particular tissue, but were not 2 fold greater than that detected in other tissues, were classified as preferential. Most of the genes predicted to encode GPI-anchored proteins (8/9) were preferentially expressed by parasites infecting SG or PV, while one appeared to be constitutively expressed during development in the tsetse host (Table 2, Figure 3). Seven of the 15 expressed genes represented 3 different gene families in the *T. brucei* genome, while 1 gene (*Tb09.211.4155*) was found to be a single copy gene conserved across the TriTryp genomes.

#### Gene expression in mammalian infective metacyclic parasites in tsetse saliva

To further characterize the 8 SG specific genes, their expression in metacyclic (MCF) parasites in saliva and parasitized SG were evaluated by RT-PCR analysis. Transcripts detected in parasitized SG could represent genes expressed by the immature SG trypanosome stages, by both the immature SG trypanosome stages and the vertebrate infective MCF parasites, or by the MCF parasites free in tsetse saliva. In contrast, transcripts detected in MCF samples specifically represent free forms in saliva. Transcripts corresponding to all 8 probable GPI-anchored proteins were detected in MCF cDNAs, representing infective trypanosomes that are injected into the mammalian host (Table 3).

**Table 3:** Salivary gland specific gene expression in metacyclic trypanosomes in saliva.

Gene Identifier	Metacyclic Transcripts
***Tb927.7.360***	+
***Tb927.7.380***	+
***Tb927.7.440***	+
***Tb927.7.400/Tb927.7.420***	+
***Tb927.6.1310***	+
***Tb927.8.950***	+
***Tb09.211.4155***	+

**+:**  = transcript detected.

Eight genes previously determined to be specifically expressed in salivary glands were analyzed from metacyclic cDNAs by RT-PCR analysis.

## Discussion

Here we report on the identification of *T. brucei* genes encoding predicted unknown surface proteins obtained via *in silico* GPI-anchor attachment signal sequence prediction analysis. Expression profiling analysis from mammalian and tsetse developmental stages indicate that transcripts for the majority of the hypothetical and hypothetical conserved proteins are expressed in parasites during their development in the tsetse salivary glands and proventriculus. Most notably, we identified 8 trypanosome genes specifically expressed in parasitized salivary glands, expression for all of which was also detected from mammalian infective MCF trypanosomes present in fly saliva. The results of this analysis give the first large-scale insight into stage-regulated expression of genes encoding putative hypothetical surface proteins during key developmental processes in the tsetse fly, and support the established paradigm of differential expression through development. Functional characterization of these unknown proteins, particularly expressed by metacyclics in saliva, ay lead the way to novel transmission blocking strategies in the mammalian host.

Proteins with GPI posttranslational modification are typically expressed on the surface of eukaryotic parasites and have the potential to participate in important biological processes such as cell–cell interactions, signal transduction, endocytosis, complement regulation, and antigenic presentation [27]. In protozoan parasites, GPI anchored glycoconjugates extensively coat the plasma membrane and are involved in many aspects of host–parasite interactions, such as adhesion and invasion of host cells, modulation and evasion from host immune response [26]. As such, there is interest in identifying the surface proteins of the medically important kinetoplastids, as reported in *L.* (*V.*) *braziliensis* and *T. cruzi* where proteomic techniques were applied to capture this class of proteins [28]–[30]. Current knowledge of the VSGs and procyclins, two of the best characterized GPI-anchored surface proteins of *T. brucei* has demonstrated the importance of these proteins in trypanosome developmental processes. Further, GPI biosynthesis has also been implicated as a molecular target for development of new drugs against African sleeping sickness [31], [32]. The availability of the *T. brucei* genome allows for postgenomic discoveries including screens for hallmark motifs such as GPI anchor attachment signals associated with surface proteins [26].

Several publically available programs can be used to predict post-translational modifications (PTM) such as glycosylation and GPI-anchor attachment, although a gold standard for prediction software remains to be found [33]. As a result, experimental validation of predicted features is always warranted. The quality of predictive algorithm outputs vary in response to several factors. In the case of GPI-anchor prediction, variables include the size of the motif recognized, quality of the underlying data used to test the algorithm, and correct application of learning procedures such as neural networks [34], [35]. The ideal tool would have high sensitivity to detect true positives, with a low false prediction rate [33]–[35]. Also relevant is the biological context being considered, as a result there are algorithms specifically for protozoa, fungi, plants, etc [34]. As seen with our dataset, two algorithms can generate different results from the same dataset. In this work, FragAnchor agreed with most, but not all of those genes previously identified by a BigPI search specific for protozoa GPI anchor attachment domains. A similar outcome with these two programs was reported after testing both against known positive and negative control GPI-anchored protein datasets [34], and against a dataset from the protozoan pathogen *Plasmodium falciparum* [19]. In both of these cases, although correct identification of true GPI-anchored proteins was high, the false positive rate was high as well. Conversely, another group found FragAnchor to be more accurate than BigPI, while maintaining the same false positive rate [35], although limitations associated with the algorithm they employed for comparison make it difficult to draw clear conclusions [34]. With these challenges in mind, we opted for a conservative approach in the identification of putative GPI-anchored proteins by selecting only those genes encoding products that showed agreement between the two predictive programs. As the absence of predicted trans-membrane domains is necessary to support a prediction of GPI-anchoring [26], we further excluded putative proteins bearing any predicted trans-membrane domains from expression analysis despite predictions of GPI-anchoring. While the presence of a GPI anchor attachment signal suggests cell surface membrane expression as mentioned earlier, there is evidence that both N- and O- glycosylation status directs nascent proteins to the apical region [35]–[37]. Like GPI anchor attachment sites, glycosylation sites can be predicted using *in silico* methodology. Importantly, while the presence of predicted glycosylation sites support the expectation of surface expression, the absence of glycosylation does not imply a lack of surface expression of a protein [38].

Fifty-six of the *in silico*-identified genes in the *T. b. brucei* genome had known or predicted functions in other closely related kinetoplastid parasites and were not pursued for further expression analysis. These included all members of the *BARP* family, and many genes with putative functions, such as *GP-63 surface protease* (5 copies), *trans-sialidase* (4 copies), *procyclin associated gene 4* (2 copies), and numerous carrier or transporter proteins. Our aim was to identify unknown SG stage-regulated genes for downstream characterization and investigation as novel transmission blocking targets. Of the 163 non-procyclin, non-VSG coding genes that were identified as encoding GPI-anchor proteins using the BigPI prediction software, 104 were confirmed with FragAnchor. With regard to possible function of these gene products, 106/163 had no known functions. A search of the available whole genome sequence information from *T. b. gambiense*, *L. major*, *T. cruzi*, *T. congolense* and *T. vivax* indicated that about 21% (22/106) of the identified genes were unique to *T. b. brucei*. With regard to the 25 genes that met our criteria to be considered likely to encode predicted GPI-anchored proteins, 5 were conserved at the level of the TriTryp genomes, 10 were shared with other species of *Trypanosoma*, and 10 were unique to *T. b. brucei*. It is possible that the lack of homologs in these genomes reflects the different biology of the parasite species, although it is also possible that as genome annotations improve homologs may be revealed. While *T. b. gambiense* is more closely related to *T. b. brucei* than the other trypanosomatid species analyzed, its biology differs from *T. b. brucei*. It remains to be seen if the unique genes in *T. b. brucei* genome contribute to its differing epidemiology. The annotated whole genome sequence of *T. b. rhodesiense* is not yet available, however, the status of *T. b. brucei* specific genes in *T. b. rhodesiense* is of interest both from an evolutionary and epidemiological point of view.

Gene expression profiling analysis showed that the majority of the 21 genes for which we detected transcripts, are expressed by trypanosome developmental stages present in the tsetse fly PV and SG tissues, while comparatively fewer are expressed by mammalian bloodstream forms and none in the MG. A similar trend was found in genes encoding proteins with less likelihood of GPI anchoring. Similarly, a proteomic analysis that identified GPI-anchored molecules in *T. cruzi* insect-stage epimastigote cultures also found the majority of the identified proteins to be novel [30]. In the case of *T. brucei*, obtaining sufficient epimastigote and metacyclic parasites from infected tsetse flies for functional analysis is difficult since these stages are unculturable *in vitro*. Confirmation of the corresponding stage-regulated protein expression is a necessary next step, and the resulting data may shed light on the roles of these products in parasite biology. Complex gene expression profiles for putative surface proteins in the proventricular and salivary gland stages of *T. brucei* may reflect the multiple discrete trypanosome developmental stages infecting these tissues, or heightened sensitivity of these trypanosomes to the tsetse or mammalian bite-site host environment. Unlike the SG and PV, far fewer unknown putative surface proteins were associated with the BSF and MG stages. This minimal detection of unknown transcripts in PF and BSF samples may be related to the abundant expression of known GPI-anchored major surface proteins in these stages- specifically the procyclins and VSGs, respectively.

Interestingly, genes encoding 8 of the 21 putative GPI-anchored proteins were specifically upregulated by parasites infecting tsetse SG. Although trypanosomes undergo four distinct developmental steps in this tissue, only two GPI-anchored protein families have been demonstrated on the surface of any SG stages to date. The alanine-rich BARP proteins are expressed on epimastigotes attached to the salivary gland epithelium. Free metacyclics in saliva no longer express BARP, but have upregulated the metacyclic variant surface glycoproteins (M-VSGs) in advance of inoculation into the mammalian host [17], [39]. The data presented here suggest a more complex series of events may be involved in the maturation of the SG-inhabiting trypanosome stages. Proteins specifically expressed on the immature SG stages might be involved in host-parasite interactions and as such could be targeted to prevent parasite maturation in the fly using genetic modification strategies in the tsetse host [40]. On the other hand, proteins expressed on the mature metacyclics may present novel vaccine targets for use in the vertebrate hosts.

Importantly, transcripts corresponding to the SG stage-regulated genes were not detected in the bloodstream form stages. Since the mammalian infective metacyclic trypomastigote is suggested to be “pre-adapted” to life in the vertebrate host, one could expect these samples to share proteins. There are two potential explanations for this observation. First, many gene products associated with adaptation to the vertebrate environment are likely to be intracellular i.e. related to energy metabolism, and therefore not bearing GPI-anchor attachment domains. As a result, these genes are expected to have been excluded from the *in silico* screen applied here. Second, when an infective fly bites the vertebrate host, metacyclic parasites are detected for several days with the bloodstream forms being not apparent until nearly a week after the infective bite [41], [42]. Thus it is possible that transitional metacyclics (t-MCFs), i.e. those detected in vertebrate blood in the days immediately after an infective tsetse bite, but before differentiation to the BSF, may have a transcriptome that reflects the parasite adaptation process from the environment of invertebrate saliva to vertebrate blood.

MCF trypanosomes, like malarial sporozoites, are the critical developmental stage of the parasite which gives rise to infection in the vertebrate host. While considerable effort has been mounted towards development of a sporozoite vaccine for the prevention of malaria, this has not been the case with the MCF of *T. brucei*. To date, VSGs have effectively thwarted all attempts at developing a vaccine against the mature BSF. It is thought that MCF parasites also express variable proteins (M-VSGs), which would hamper vaccine development efforts targeting MCF. Our results suggest however that GPI-anchored surface protein repertoire of MCF may be more complex and different from the BSF forms than originally thought. The expression of the genes encoding putative surface proteins on the mammalian-infective stage suggests a complex interface of MCF and mammalian bite-site.

In summary, the *in silico* and semi-quantitative gene expression analyses approach used here has allowed an important first look at the stage-regulated expression of genes encoding putative GPI-anchored proteins with no known functions in the human and animal pathogen *T. brucei*. The findings presented here suggest that the tsetse host-parasite interplay during differentiation may be quite complex. Most importantly, these results greatly increase our understanding of trypanosome biology at the point of transmission to the vertebrate host, and identify a number of putative invariant surface proteins, which could be investigated further for novel transmission blocking strategies.

## Supporting Information

Table S1 Gene specific primer pairs sequence used for RT-PCR analysis*.
(DOC)

Table S2 Genes that were excluded from expression analysis.
(DOC)

Table S3 Gene specific qRT-PCR primer sequences used for expression data validation. qRT-PCR primers were designed by Beacon Designer™ software (Premier Biosoft International, Palo Alto, California), or the OligoPerfect™ Designer primer design tool. qRT-PCR cycling conditions were: 95°C for 8 min, 40 cycles (95°C for 15 s, 30 s annealing, 72°C for 30 s), 95°C for 1 m, and 55°C for 1 m. Annealing temperatures were adjusted for each primer pair combination as shown.
(DOC)

Table S4 Gene products with known or predicted functions identified by an *in silico* screen of the first published genome of *T. brucei* using BigPI software (genes encoding VSG and ESAG filtered out). The gene set was re-evaluated using a second algorithm, FragAnchor, and agreement between the algorithms is noted.
(DOC)

Table S5 Interpro domains detected from the 106 putative gene products initially identified as encoding predicted GPI-anchored protein retrieved from TriTrypDB. The location of the domain within the CDS of the gene is listed. Genes from which no domain was detected are excluded from this list.
(DOC)

Table S6 Bioinformatic analyses of the 81 putative genes that had a lower likelihood of encoding GPI-anchored proteins. These gene products either had TM domains, lacked signal peptide domains, or were predicted by only one of the GPI anchor prediction analysis. Forty-nine genes were conserved at the TriTryp level, having orthologs in the *T. cruzi* and *L. major* genomes. Twenty-one gene products had homologs identified in the available genome sequences of other species of *Trypanosoma* (*T. cruzi*, *T. congolense*, *T. vivax*, or *T. b. gambiense*) but not in *L. major*. Finally 12 genes examined were found to be present only in the genome of *T. b. brucei*, but not in any related kinetoplastid.
(DOC)

Table S7 Developmental stage regulation and level of expression of gene products that were predicted to be less likely to contain GPI- anchor attachment signal domains.
(DOC)

## References

[1] Morrison LJ, Marcello L, McCulloch R (2009). Antigenic variation in the African trypanosome: molecular mechanisms and phenotypic complexity.. Cell Microbiol.

[2] Aksoy S, Gibson WC, Lehane MJ (2003). Interactions between tsetse and trypanosomes with implications for the control of trypanosomiasis.. Adv Parasitol.

[3] Sharma R, Gluenz E, Peacock L, Gibson W, Gull K (2009). The heart of darkness: growth and form of *Trypanosoma brucei* in the tsetse fly.. Trends Parasitol.

[4] Berriman M, Ghedin E, Hertz-Fowler C, Blandin G, Renauld H (2005). The genome of the African trypanosome *Trypanosoma brucei*.. Science.

[5] El-Sayed NM, Myler PJ, Bartholomeu DC, Nilsson D, Aggarwal G (2005). The genome sequence of *Trypanosoma cruzi*, etiologic agent of Chagas disease.. Science.

[6] Ivens AC, Peacock CS, Worthey EA, Murphy L, Aggarwal G (2005). The genome of the kinetoplastid parasite, *Leishmania major*.. Science.

[7] Peacock CS, Seeger K, Harris D, Murphy L, Ruiz JC (2007). Comparative genomic analysis of three *Leishmania* species that cause diverse human disease.. Nat Genet.

[8] Jackson AP, Sanders M, Berry A, McQuillan J, Aslett MA (2010). The genome sequence of *Trypanosoma brucei gambiense*, causative agent of chronic human African trypanosomiasis.. PLoS Negl Trop Dis.

[9] Kolev NG, Franklin JB, Carmi S, Shi H, Michaeli S (2010). The transcriptome of the human pathogen *Trypanosoma brucei* at single-nucelotide resolution.. PLoS Pathogens.

[10] Ferguson MA (1999). The structure, biosynthesis and functions of glycosylphosphatidylinositol anchors, and the contributions of trypanosome research.. J Cell Sci.

[11] Chatterjee S, Mayor S (2001). The GPI-anchor and protein sorting.. Cell Mol Life Sci.

[12] Ruepp S, Furger A, Kurath U, Renggli CK, Hemphill A (1997). Survival of *Trypanosoma brucei* in the tsetse fly is enhanced by the expression of specific forms of procyclin.. J Cell Biol.

[13] Vassella E, Butikofer P, Engstler M, Jelk J, Roditi I (2003). Procyclin null mutants of *Trypanosoma brucei* express free glycosylphosphatidylinositols on their surface.. Mol Biol Cell.

[14] Nolan DP, Jackson DG, Biggs MJ, Brabazon ED, Pays A (2000). Characterization of a novel alanine-rich protein located in surface microdomains in *Trypanosoma brucei*.. J Biol Chem.

[15] Urwyler S, Studler E, Renggli CK, Roditi I (2007). A family of stage-specific alanine-rich proteins on the surface of epimastigote forms of *Trypanosoma brucei*.. Mol Microbiol.

[16] Stephens NA, Hajduk SL (2011). Endosomal localization of the Serum Resistance-Associated protein in African trypanosomes confers human infectivity.. Eukaryot Cell.

[17] Steverding D, Stierhof YD, Chaudhri M, Ligtenberg M, Schell D (1994). ESAG 6 and 7 products of *Trypanosoma brucei* form a transferring binging protein complex.. Eur J Cell Biol.

[18] Eisenhaber B, Bork P, Eisenhaber F (1999). Prediction of potential GPI-modification sites in proprotein sequences.. J Mol Biol.

[19] Poisson G, Chauve C, Chen X, Bergeron A (2007). FragAnchor a large scale all Eukaryota predictor of Glycosylphosphatidylinositol-anchor in protein sequences by qualitative scoring.. Genomics Proteomics Bioinformatics.

[20] Petersen TN, Brunak S, von Heiine G, Nielsen H (2011). SignalP 4.0: discriminating signal peptides from transmembrane regions.. Nature Methods.

[21] Cserzo M, Wallin E, Simon I, von Heijne G, Elofsson A (1997). Prediction of transmembrane alpha-helices in procariotic membrane proteins: the Dense Alignment Surface method.. Protein Eng.

[22] Julenius K, Mølgaard A, Gupta R, Brunak S (2005). Prediction, conservation analysis and structural characterization of mammalian mucin-type O-glycosylation sites.. Glycobiology.

[23] Lanham SM (1968). Separation of trypanosomes from the blood of infected rats and mice by anion-exchangers.. Nature.

[24] Lanham SM, Godfrey DG (1970). Isolation of salivarian trypanosomes from man and other mammals using DEAE-cellulose.. Exp Parasitol.

[25] Moloo SK (1971). An artificial feeding technique for *Glossina*.. Parasitology.

[26] Acosta-Serrano A, Hutchinson C, Nakayasu ES, Almeida IC, Carrington M, Barry D, McCulloch R, Mottram J, Acosta-Serrano A (2007). Comparison and evolution of the surface architecture of trypanosomatid parasites.. Trypanosomes After the Genome.

[27] Paulick MG, Bertozzi CR (2008). The glycosylphosphatidylinositol anchor: A complex membrane-anchoring structure for proteins.. Biochemistry.

[28] Rojas A, Garcia-Lugo P, Crisante G, Anez-Rojas N, Anez N (2008). Isolation, purificiation, characterization and antigenic evaluation of GPI-anchored membrane proteins from *Leishmania* (*Viannia*) *braziliensis*.. Acta Trop.

[29] Cordero EM, Nakayasu ES, Gentil LG, Yoshida N, Almeida IC (2009). Proteomic analysis of detergent-solubilized membrane proteins from insect-developmental forms of *Trypanosoma cruzi*.. J Proteome Res.

[30] Nakayasu ES, Yashunsky DM, Nohara LL, Torrecilhas ACT, Nikolaev AV (2009). GPIomics: global analysis of glycosylphosphatidylinositol-anchored molecules of *Trypanosoma cruzi*.. Mol Syst Biol.

[31] Smith TK, Crossman A, Brimacombe JS, Ferguson MA (2004). Chemical validation of GPI biosynthesis as a drug target against African sleeping sickness.. EMBO J.

[32] Kinoshita T (2008). Designing sleeping sickness control.. ACS Chem Biol.

[33] Blom N, Sicheritz-Ponten T, Gupta R, Gammeltoft S, Brunak S (2004). Preditction of post-translational glycosylation and phosphorylation of proteins from the amino acid sequence.. Proteomics.

[34] Eisenhaber B, Eisenhaber F (2010). Prediction of posttranslational modification of proteins from their amino acid sequence.. Methods Mol Biol.

[35] Pierleoni A, Martelli PL, Casadio R (2008). PredGPI: a GPI-anchor predictor.. BMC Bioinformatics.

[36] Pang S, Urquhart P, Hooper NM (2004). N-glycans, not the GPI-anchor, mediate the targeting of a naturally glycosylated, GPI-anchored protein in polarized epithelial cells.. J Cell Sci.

[37] Urquhart P, Pang S, Hooper NM (2005). N-glycans as apical targeting signals in polarized epithelial cells.. Biochem Soc Symp.

[38] Weisz OA, Rodriguez-Boulan E (2009). Apical trafficking in epithelial cells: signals, clusters and motors.. J Cell Sci.

[39] Vickerman K (1985). Developmental cycles and biology of pathogenic trypanosomes.. Br Me Bull.

[40] Aksoy S, Maudlin I, Dale C, Robinson AS, O'Neill S (2001). Prospects for control of African trypanosomiasis by tsetse vector manipulation.. Trends Parasitol.

[41] Esser KM, Schoenbechler MJ, Gingrich JB (1982). *Trypanosoma rhodesiense* blood forms express all antigen specificities relevant to protection against metacyclic (insect form) challenge.. J Immunol.

[42] Lenardo MJ, Rice-Ficht AC, Kelly G, Esser KM, Donelson JE (1984). Characterization of the genes specifying two metacyclic variable antigen types in *Trypanosoma brucei rhodesiense*.. Proc Natl Acad Sci U S A.

