# Supplementary material for: Transcript Expression Analysis of Putative Trypanosoma brucei GPI-Anchored Surface Proteins during Development in the Tsetse and Mammalian Hosts
Source: PLoS Negl Trop Dis. 2012 Jun 19;6(6):e1708. doi: 10.1371/journal.pntd.0001708 (PMC3378594; doi:10.1371/journal.pntd.0001708)
Supplement: Table S1 — Gene specific primer pairs sequence used for RT-PCR analysis*. (DOC) [file pntd.0001708.s001.doc]

| **Primer Name** | **PCR Primer Sequence** |
| --- | --- |
| *TubulinF* | CTCGACACACTCACTTCTGGAG |
| *TubulinR* | CGAATTTGTGGTCAATACGAG |
| *BARP* | GAAGCAAAGGTACAAGCAG |
| *BARP* | GATGCCAACTGTCGTAAACTC |
| *Tb927.3.2400F* | GTAATCAGTTTCGGCCTCTACG |
| *Tb927.3.2400R* | GGTGGGATAGGAGAACAAAGTG |
| *Tb11.02.4230F* | CCTTAGCTTCTGCCCTGTTG |
| *Tb11.02.4230R* | GACGTCCACCAGTCTTTCCAC |
| *Tb927.1.4220F* | GGCACCTGGTAGAAGTGAGC |
| *Tb927.1.4220R* | GTGCAGGACATTGTGCAAG |
| *Tb927.7.3570F* | GAGGTGGTAATGTCAGCAAC |
| *Tb927.7.3570R* | GTGAAAGCGCCATTTCTAGTCT |
| *Tb927.8.1250F* | CTCGGTCAATTGTATGCCAC |
| *Tb927.8.1250R* | CACTTGGATCACGGTACCAC |
| *Tb927.7.6370F* | GGATCGCCCTATGTGCTTTAGG |
| *Tb927.7.6370R* | GAGGTGGCATCGTAGTGGATAC |
| *Tb927.4.1110F* | GATCATTGTGCTCAACACTGTCC |
| *Tb927.4.1110R* | CTAGATAAATTCCCGACGGTTG |
| *Tb927.1.1500F* | CCCAGATGTCTGTATTCGTTG |
| *Tb927.1.1500R* | GGAAGAGGAAGTGATACGCAG |
| *Tb927.10.990F* | GTAGAACGGATGAAGGGAATTG |
| *Tb927.10.990R* | GGCAGGAAGTATTAACCAAACG |
| *Tb09.211.0010F* | GTGATGAGTTGGAACGAAGAC |
| *Tb09.211.0010R* | CATTTCCTCCTGTAGCTCCTG |
| *Tb927.1.530F* | CAACTGTTTGGACGGTAGATG |
| *Tb927.1.530R* | GACCTCATGAGAGAACGAAG |
| *Tb927.10.12060F* | CGCACCACTACTAACACTGAGC |
| *Tb927.10.12060R* | CCTGCTTCTCTATATCGTGTTCC |
| *Tb927.4.3270F* | GTGGTGGTATGTGCTTACGG |
| *Tb927.4.3270R* | GGTAGAACTCTTTGCCCAG |
| *Tb927.4.3290F* | GCCTGCGAATCTAACGAGTATC |
| *Tb927.4.3290R* | CCATTTCCACCAGGTACTTCTC |
| *Tb927.3.1200F* | CACTTTCCCTAACCGGTAGTTGG |
| *Tb927.3.1200R* | GCTGTCTGTAGTTCAGGCAAACG |
| *Tb09.v1.0450F* | GGCTTTCTTTGACAGTTCGAGG |
| *Tb09.v1.0450R* | CTGTGCTGCAGTAAGATTGAGC |
| *Universal360F* | GTCATGTAAGTGCGGCATGTCTC |
| *Universal360R* | CTCTCGCAATTCGTCTTGCAG |
| *Tb927.4.3250F* | GACCGAAAACGAGGAATAG |
| *Tb927.4.3250R* | CTCCAACTTCTTCCTCAAACC |
| *Tb09.211.3340F* | GGAATTCAGTGAAAAGGTGGAG |
| *Tb09.211.3340R* | GCAGTTCCACCTGCAGCATTTAC |
| *Tb927.6.1310F* | GAAGACGAAGTGCAGAGTCATG |
| *Tb927.6.1310R* | CAGCTCCAGATTCCTTTAATGC |
| *Tb09.v1.0470F* | GATTACAGTGGTTACGGGAGGAG |
| *Tb09.v1.0470R* | CCTCCTCATGTAAGATAACATTGG |
| *Tb11.01.2940F* | GGTGTGTTCGCGACGTGCGACGC |
| *Tb11.01.2940R* | CATCACCAACAAGCAATGAAGTG |
| *Tb927.7.5300F* | GTACCCTCCACCTCTGGACAATC |
| *Tb927.7.5300R* | GCAAGCAACTCTTCAGGGTACTC |
| *Tb927.5.3260F* | GCCAATGATGGTCTTGCATCTG |
| *Tb927.5.3260R* | GCACTGCACCAACTACTGAAG |
| *Tb09.211.4155F* | CTATGGCCATCTTATCCGAGAG |
| *Tb09.211.4155R* | CACAATCACCGCTGTGTTAATG |
| *Tb927.4.570F* | GACTATTCGACAGGTGGTCACAGC |
| *Tb927.4.570R* | CATTGCCAACTTTCCTTCCGCATG |
| *Tb09.211.2750F* | CGCGTTTAAAGGGTTCATACTTG |
| *Tb09.211.2750R* | CGTGAAGTGTCATCGCTAATGG |
| *Tb927.4.3210/3230F* | GCCATCTGTAGTATAGTTAGG |
| *Tb927.4.3210/3230R* | GTTCCAGAAGCAACCATACGAG |
| *Tb09.v1.0500/0530F* | GAGAGGAGGGCGTCGATGATGC |
| *Tb09.v1.0500/0530R* | GGTTATAAGCATAAGCACCATC |
| *Tb927.10.5770/5710F* | CTGTTTCTTCGCAACCCAGCGG |
| *Tb927.10.5770/5710R* | CTTACCGTCCTTCACATCTTCC |
| *Tb11.01.7980F* | CAGAAAGATTACGCTCGTGTTG |
| *Tb11.01.7980R* | CACCGACAAATTATTGTGAGCG |
| *Tb09.211.2460F* | CTAATGCAAGATGCGGTATCATC |
| *Tb09.211.2460R* | GTCTCCATCCACTCCGTTAAAGTG |
| *Tb927.10.8930F* | GGTCTGCAGAACTCTGCAAGG |
| *Tb927.10.8930R* | GTCAAGTGCACATATCGGATG |
| *Tb927.10.2820F* | CCAAGTCATCTATCGGTGTTG |
| *Tb927.10.2820R* | CGCTGGGAGCAGATACTTTTCG |
| *Tb11.01.8300F* | GTGTACAGGGTGAGCAGAAAC |
| *Tb11.01.8300R* | GAATGATTGGCTCACCGAACGC |
| *Tb927.10.4010F* | CATGTGAGGAGGATGTTGTTCGC |
| *Tb927.10.4010R* | CATCCACACTAACAGCCACACTAC |
| *Tb927.5.2970F* | CAAGGTGTTACGTGCTGTTGATG |
| *Tb927.5.2970R* | GGAGTGGCATTGGTAAAAGAAG |
| *Tb11.02.1920F* | GAAGGAAGTTACCTGCATCGTG |
| *Tb11.02.1920R* | CGAGCTGAACTCCATCTGAATC |
| *Tb927.4.2940F* | CAGTGGCTCCTCATTACCTC |
| *Tb927.4.2940R* | CGTGTCTTCCACTTCCTACCG |
| *Tb927.3.1660F* | GATACGATAACGCAAGGGAAG |
| *Tb927.3.1660R* | GGTCCTTGCACAGTACCGTATTC |
| *Tb10.61.1700F* | GCACCTTCGTACCACACTTTCG |
| *Tb10.61.1700R* | GCAGTGCATAATAGTGTAACG |
| *Tb927.10.7480F* | CTATATGGAACGAAATGCTCGAC |
| *Tb927.10.7480R* | CAGAACAACGTTGGGTAAGAC |
| *Tb927.6.1070F* | CTCGAGTGCCTTATTGAGTGTG |
| *Tb927.6.1070R* | CGAAGGAGAGGTACGAAATGAC |
| *Tb09.211.4710F* | GACATTGTGGTACCCGACTCTTG |
| *Tb09.211.4710R* | CACAGCACTCACTGAGGGATAC |
| *Tb927.5.1960F* | GAAATTGAGTTAACCCGCAGTC |
| *Tb927.5.1960R* | GCAGCCACATTATCGTTGTTG |
| *Tb927.7.4350F* | GCAGTTGTTGGAAGATTCTCGC |
| *Tb927.7.4350R* | CCTGAGCCTGAATGACCTTTAC |
| *Tb09.211.4690F* | GCGTCTTTCCTAGTTCCTGTG |
| *Tb09.211.4690R* | GAAGTGCGATATAGTGCCAAC |
| *Tb11.02.2230F* | CACCTCAACTTGTAGCAGCATC |
| *Tb11.02.2230R* | CGTCGTCAAATAGACGGATCAC |
| *Tb927.10.9290F* | GAGGAATAAAGCGGTAATGCAC |
| *Tb927.10.9290R* | CGTCATCTCACGCATTATGTC |
| *Tb11.01.4650F* | CATGCTCAACACAACAGTGAAG |
| *Tb11.01.4650R* | GAACCCAATAACACCAACGTTTC |
| *Tb927.7.4630F* | GTGCTCGGTCCTACTTCTTCAG |
| *Tb927.7.4630R* | CATTCCAAGAATCAATAGCGAG |
| *Tb927.3.2780F* | CCGCACGGAGTGGGAACAGC |
| *Tb927.3.2780R* | GAGGCACAACTCTTCCAATACC |
| *Tb11.02.1540F* | CTCTACACACCCACCATCAG |
| *Tb11.02.1540R* | CAATGCTTTCCCACACTATC |
| *Tb927.4.5120F* | GAAGAGGTTAAGCGCATGAAG |
| *Tb927.4.5120R* | CTGAGAGCCTGTACATCGGTAAG |
| *Tb927.10.11290F* | GACTCTCTGGTGGGTGAGTTG |
| *Tb927.10.11290R* | GCTCACACAGGGTAATCACAGG |
| *Tb927.4.4550F* | GCCCTCCAGTGAACTATACGAG |
| *Tb927.4.4550R* | CGCTCTAAGTCCAGATCTTCC |
| *Tb927.4.3440F* | CTAGCGTTTCCTCCTAGACTGC |
| *Tb927.4.3440R* | CAGTACTTCATGCACACTGCAAG |
| *Tb927.8.930F* | CAGAGGCCTAAAAGGTGCAG |
| *Tb927.8.930R* | TGTGGTTCTTATTGCTTGCTG |
| *Tb927.8.950F* | CAGAGGCCTAAAAGGTGCAG |
| *Tb927.8.950R* | CGACCTTCACCAAACTCGAT |
| *Tb927.10.4390F* | TGCAATTTCGTGAAAACCAA |
| *Tb927.10.4390R* | AAAGTGGCATGTGTTGGTGA |
| *Tb927.10.4380F* | TGGAAGAGGAAATGTGGTCCAA |
| *Tb927.10.4380R* | AAAGTGGCATGTGTTGGTGAC |
| *Tb06.3A7.960F* | GCAGGGTGTGAAGGAAGGTA |
| *Tb06.3A7.960R* | AACAGTAACGCCGACTGTCC |
| *Tb11.02.1565F* | CATCGGTGCTCAGTTTTGAA |
| *Tb11.02.1565R* | ACAACGGTCACCGAATAAGG |
| *Tb10.70.4020F* | ATGCAACACTTTGTTGGTGG |
| *Tb10.70.4020R* | TTAAGTGGCAAATACATTTACACTTAA |
| *Tb10.70.0330F* | GTTTGGGTCGAAATCACTGG |
| *Tb10.70.0330R* | GCCTTTGCTGAAAGAGGTTG |
| *Tb09.211.4070F* | ATCCGCCTGACGTAGAAGAA |
| *Tb09.211.4070R* | ATCGCGGAAACTAAGAGCAG |
| *Tb927.3.2800F* | TAGCCAGAGCCCTCATCACT |
| *Tb927.3.2800R* | GCACTACCCGAATGCGTAAT |
| *Tb927.5.4020F* | TATGTGAGGTGGCGAAACAA |
| *Tb927.5.4020R* | TGCATGTACAGCCCTCTCAG |
| *Tb11.02.0390F* | GGCACTTGGTGTGTTTCCTT |
| *Tb11.02.0390R* | TTACGACAGCAGCACACCTC |
| *Tb927.5.4010F* | TGATGGGCAAGAGAAGGAAG |
| *Tb927.5.4010R* | GCGATTTAATACCGCCATCT |
| *Tb10.6k15.3520F* | TTCCGTTATTTCCTGCAACC |
| *Tb10.6k15.3520R* | TGTGCAGTCCTGAAAACAGC |
| *Tb927.8.7310F* | TGACGAAGTGTGCAGAAAGG |
| *Tb927.8.7310R* | CTGTGGCTCAGTCGATTTCA |
| *Tb11.02.2370F* | GTGCCATTCCTGGAAGATGT |
| *Tb11.02.2370R* | CGTAATGGTGGCCAATAACC |
| *Tb927.3.1230F* | CAGTTGCCCTTTTTCTCAGC |
| *Tb927.3.1230R* | CATCGGGCACCTTCACTATT |
| *Tb927.8.7330F* | CCTCGTTGAAGTGACGGTTT |
| *Tb927.8.7330R* | GGTTACTCTGCGGCTCAGTC |
| *Tb10.70.3780F* | ATAGCGTGGAACGGAATTTG |
| *Tb10.70.3780R* | GGCACTAATCAGCGACAACA |
| *Tb927.8.490F* | CTGCTGAAGCCTCTTTTTGG |
| *Tb927.8.490R* | CGTCGTCTTCCGTCCATAAT |
| *Tb09.142.0410F* | AATCTAGCGCCGGTGTTTC |
| *Tb09.142.0410R* | CCAAACTCGAGAGCACAACA |

* Parameters specified in the primer design software were oligos which had a 50% GC:AT ratio, 60°C melting temperature, and 21-26 bp in length. Possible primer pair sequences identified by the algorithm were individually evaluated. Where possible, they were adjusted or selected to maintain a high GC content, the presence of G or C at the 5’ and/or 3’ termini of the oligo, and minimal nucleotide repeats. The primer sequences were analyzed against the *G. morsitans* (http://www.sanger.ac.uk/Projects/G_morsitans) and *T. brucei* (<http://www.genedb.org/Homepage/Tbruceibrucei927>) genomic DNA sequences using the BLAST search option to confirm specificity to the targeted gene. To confirm that the primers generated the expected size gene product, *T. b. brucei* gDNA was used as the template in a PCR amplification reaction. As a positive control, trypanosome *alpha tubulin* (*Tubulin*) specific primers were used. Any primer pair not yielding the expected size product or generating multiple bands was removed from experimental analyses. In the case of three pairs of genes, nucleotide sequences were too similar to design primers that could differentiate between them. In these cases, primers were expected to amplify both members of the pair and this was denoted in the primer identifier.
