# Supplementary material for: Transcript Expression Analysis of Putative Trypanosoma brucei GPI-Anchored Surface Proteins during Development in the Tsetse and Mammalian Hosts
Source: PLoS Negl Trop Dis. 2012 Jun 19;6(6):e1708. doi: 10.1371/journal.pntd.0001708 (PMC3378594; doi:10.1371/journal.pntd.0001708)
Supplement: Table S2 — Genes that were excluded from expression analysis. (DOC) [file pntd.0001708.s002.doc]

| **Hypothetical Conserved** | | **Hypothetical** | |
| --- | --- | --- | --- |
| **Tb ORF** | **Exclusion Criteria**^*^ | **Tb ORF** | **Exclusion Criteria**^*^ |
| *Tb09.v1.0500/ Tb09.v1.0530* | cDNA | *Tb11.02.1565* | gDNA |
| *Tb927.4.3290* | cDNA | *Tb927.10.3410* | cDNA |
| *Tb11.01.8300* | no correlation | *Tb927.8.7310* | gDNA |
| *Tb927.10.4010* | no correlation |  |  |
| *Tb927.3.1660* | cDNA |  |  |
| *Tb09.211.3340* | cDNA |  |  |
| *Tb927.7.5300* | cDNA |  |  |
| *Tb927.5.3260* | cDNA |  |  |
| *Tb09.v1.0470* | cDNA |  |  |
| *Tb927.7.3570* | cDNA |  |  |
| *Tb927.1.530* | cDNA |  |  |
| *Tb927.4.3440* | no correlation |  |  |
| *Tb927.7.4630* | cDNA |  |  |
| *Tb11.02.1540* | gDNA |  |  |
| *Tb927.1.1500* | cDNA |  |  |
| *Tb927.4.5120* | cDNA |  |  |
| *Tb11.02.0390* | cDNA |  |  |
| *Tb927.10.7170* | cDNA |  |  |

^*^ cDNA= no product was observed after PCR analysis of cDNA; gDNA= no product was observed after PCR analysis of gDNA; no correlation= expression profiles at 32 and 36 cycles did not match; multiple bands= more than one band was generated from one or more individual experimental cDNAs.
