# Supplementary material for: Transcript Expression Analysis of Putative Trypanosoma brucei GPI-Anchored Surface Proteins during Development in the Tsetse and Mammalian Hosts
Source: PLoS Negl Trop Dis. 2012 Jun 19;6(6):e1708. doi: 10.1371/journal.pntd.0001708 (PMC3378594; doi:10.1371/journal.pntd.0001708)
Supplement: Table S3 — Gene specific qRT-PCR primer sequences used for expression data validation. qRT-PCR primers were designed by Beacon Designer™ software (Premier Biosoft International, Palo Alto, California), or the OligoPerfect™ Designer primer design tool. qRT-PCR cycling conditions were: 95°C for 8 min, 40 cycles (95°C for 15 s, 30 s annealing, 72°C for 30 s), 95°C for 1 m, and 55°C for 1 m. Annealing temperatures were adjusted for each primer pair combination as shown. (DOC) [file pntd.0001708.s003.doc]

| **Primer Name** | **qRT-PCR Primer Sequence** | **Annealing Temperature (C)** |
| --- | --- | --- |
| *TbTubulin-1F* | GGCTTCAAGTGCGGTATC | 55 |
| *TbTubulin-1R* | GTGGAGTTGGCGATCATG |  |
| *TbTubulin-2F* | ACTCACTTCTGGAGCACACCG | 55 |
| *TbTubulin-2R* | GCTGTCAGCGAGGAAACCAC |  |
| *Tb927.8.950F* | AAGCAAAGAAGGCTGCTGAG | 55 |
| *Tb927.8.950R* | CGTGTGTCACTCTCCAGTCG |  |
| *Tb927.3.2400F* | ACGCACTACATTGAACCAAC | 53 |
| *Tb927.3.2400R* | CCACCACGGCTGAAGTAG |  |
| *Tb927.5.4020F* | GCATTGGAGGAAAGCAAAAA | 55 |
| *Tb927.5.4020R* | CGTACAGATGCCTCATACGC |  |
| *Tb927.10.5700/5710F* | CGTTGGTCATTACATCTGTCATT | 55 |
| *Tb927.10.5700/5710R* | GGAAGTGTCAGCAGCATCTAG |  |
