# Supplementary material for: Transcript Expression Analysis of Putative Trypanosoma brucei GPI-Anchored Surface Proteins during Development in the Tsetse and Mammalian Hosts
Source: PLoS Negl Trop Dis. 2012 Jun 19;6(6):e1708. doi: 10.1371/journal.pntd.0001708 (PMC3378594; doi:10.1371/journal.pntd.0001708)
Supplement: Table S4 — Gene products with known or predicted functions identified by an in silico screen of the first published genome of T. brucei using BigPI software (genes encoding VSG and ESAG filtered out). The gene set was re-evaluated using a second algorithm, FragAnchor, and agreement between the algorithms is noted. (DOC) [file pntd.0001708.s004.doc]

| **Tb ORF** | **GPI Anchor Prediction** | | **Annotation** |
| --- | --- | --- | --- |
|  | **BigPI** | **FragAnchor** |  |
| *Tb09.244.2410* | x | x | BARP protein |
| *Tb09.244.2420* | x | x | BARP protein |
| *Tb09.244.2430* | x | x | BARP protein |
| *Tb09.244.2440* | x | x | BARP protein |
| *Tb09.244.2510* | x | x | BARP protein |
| *Tb09.244.2520* | x | x | BARP protein |
| *Tb09.244.2470* | x | x | BARP protein |
| *Tb09.244.2490* | x | x | BARP protein |
| *Tb09.244.2500* | x | x | BARP protein |
| *Tb09.244.2460* | x | x | BARP protein |
| *Tb09.244.2480* | x | x | BARP protein |
| *Tb09.244.2450* | x | x | BARP protein |
| *Tb09.244.2400* | x | x | BARP protein |
| *Tb09.244.2530* | x | x | BARP protein |
| *Tb927.10.10620* | x | x | EP1 procyclin |
| *Tb927.10.10250* | x | x | EP2 procyclin |
| *Tb927.6.450* | x | x | EP3-2 procyclin |
| *Tb927.6.480* | x | x | EP3-2 procyclin |
| *Tb927.6.520* | x | x | EP3-2 procyclin |
| *Tb11.02.1100* | x | x | NT8.1 nucleobase/nucleoside transporter 8.1 |
| *Tb11.02.1105* | x | x | NT8.1 nucleobase/nucleoside transporter 8.1 |
| *Tb11.02.1106* | x | x | NT8.1 nucleobase/nucleoside transporter 8.1 |
| *Tb11.02.5610* | x | x | MSP-A, putative |
| *Tb11.02.5630* | x | x | MSP-A, putative |
| *Tb11.02.5640* | x | x | MSP-A, putative |
| *Tb927.8.1610* | x | x | MSP-B, putative |
| *Tb11.12.0006* | x | x | major surface protease gp63, putative |
| *Tb927.8.7340* | x | x | trans-sialidase, putative, neuraminidase, putative |
| *Tb927.8.7350* | x | x | trans-sialidase, putative, neuraminidase, putative |
| *Tb11.01.3240* | x | x | trans-sialidase, putative |
| *Tb927.7.6830* | x | x | trans-sialidase, putative |
| *Tb927.10.10210* | x | x | procyclin-associated gene 4 (PAG4) protein |
| *Tb11.01.6220* | x | x | procyclin-associated gene 4 (PAG4) protein, putative |
| *Tb927.3.960* | x |  | protein transport protein Sec61 gamma subunit, putative |
| *Tb927.6.4990* | x |  | ATP synthase, epsilon chain, putative |
| *Tb927.8.8040* | x | x | kinetoplastid-specific phospho-protein phosphatase, putative |
| *Tb927.6.1960* | x | x | glycosyltransferase family 28 protein, putative |
| *Tb11.42.0002* | x | x | cation transporter protein, putative |
| *Tb927.7.4980* | x |  | ZC3H23 hypothetical protein, conserve, zinc finger family member, putative |
| *Tb11.01.1320* | x |  | oxidoreductase, putative |
| *Tb927.3.5570* | x |  | syntaxin, putative |
| *Tb927.8.3330* | x | x | mitochondrial carrier protein, putative |
| *Tb927.8.2210* | x |  | PTR1 pteridine reductase |
| *Tb11.12.0013* | x |  | target SNARE, putative |
| *Tb927.10.5110* | x |  | KREPA4 |
| *Tb927.8.1820* | x |  | nonspecific lipid-transfer protein, putative, sterol carrier protein, putative |
| *Tb11.01.6090* | x |  | RPB7 RNA polymerase subunit, putative |
| *Tb927.8.1830* | x |  | tRNA-methyl transferase, putative |
| *Tb09.160.4180* | x | x | 28G16.380 inositol/phosphatidylinositol phosphatase, putative |
| *Tb927.3.3070* | x | x | PDEC 3', 5'-cyclic nucleotide phosphodiesterase, putative |
| *Tb927.4.1020* | x | x | serine-palmitoyl-CoA transferase, putative |
| *Tb927.3.4190* | x | x | endosomal integral membrane protein, putative |
| *Tb927.2.3910* | x |  | 28H13.305 beta-ketoacyl synthase family protein, putative |
| *Tb927.6.510* | x |  | GPEET2 procyclin precursor |
| *Tb11.02.3210* | x | x | TIM triosephosphate isomerase |
| *Tb09.160.3510* | x |  | 28G16.70 presenilin-like aspartic peptidase, putative |
| *Tb927.6.440* | x | x | HpHb receptor |
