# Supplementary material for: Transcript Expression Analysis of Putative Trypanosoma brucei GPI-Anchored Surface Proteins during Development in the Tsetse and Mammalian Hosts
Source: PLoS Negl Trop Dis. 2012 Jun 19;6(6):e1708. doi: 10.1371/journal.pntd.0001708 (PMC3378594; doi:10.1371/journal.pntd.0001708)
Supplement: Table S5 — Interpro domains detected from the 106 putative gene products initially identified as encoding predicted GPI-anchored protein retrieved from TriTrypDB. The location of the domain within the CDS of the gene is listed. Genes from which no domain was detected are excluded from this list. (DOC) [file pntd.0001708.s005.doc]

| **Tb ORF** | **Interpro ID** | **Description** | **Start of Match** | **End of Match** | **E-value of Match** |
| --- | --- | --- | --- | --- | --- |
| Tb09.160.0430 | IPR007498 | Paraquat-inducible protein A | 384 | 576 | 1.90E-02 |
| Tb09.211.3340 | IPR012336 | Thioredoxin-like | 9 | 129 | 5.60E-11 |
| Tb09.211.4155 | IPR013880 | Yos1-like | 5 | 99 | 9.70E-52 |
| Tb11.01.2940 | IPR019781 | WD domain, G-beta repeat | 194 | 232 | 1.20E-04 |
| Tb11.01.2940 | IPR019782 | Trp-Asp (WD) repeats profile | 200 | 232 | 1.10E+01 |
| Tb11.01.2940 | IPR017986 | Trp-Asp (WD) repeats circular profile | 200 | 241 | 1.00E+01 |
| Tb11.01.2940 | IPR001680 |  | 17 | 55 | 3.10E+00 |
| Tb11.01.2940 | IPR001680 |  | 64 | 102 | 1.40E+01 |
| Tb11.01.2940 | IPR001680 |  | 193 | 232 | 1.00E-02 |
| Tb11.01.2940 | IPR011046 | WD40 repeat-like | 21 | 323 | 1.00E-24 |
| Tb11.01.8300 |  | TPR-like | 227 | 574 | 7.50E-09 |
| Tb11.02.1540 |  | I/LWEQ domain | 344 | 414 | 1.40E-02 |
| Tb11.02.4230 | IPR020474 | Leucine-rich repeat signature | 316 | 329 | 2.90E-02 |
| Tb11.02.4230 | IPR020474 | Leucine-rich repeat signature | 548 | 561 | 2.90E-02 |
| Tb11.02.4230 | IPR003590 |  | 285 | 312 | 8.20E-02 |
| Tb11.02.4230 | IPR003590 |  | 313 | 362 | 1.20E+02 |
| Tb11.02.4230 | IPR003590 |  | 422 | 449 | 3.30E-03 |
| Tb11.02.4230 | IPR003590 |  | 450 | 477 | 4.60E-01 |
| Tb11.02.4230 | IPR003590 |  | 520 | 547 | 7.50E+01 |
| Tb11.02.4230 | IPR003590 |  | 548 | 576 | 2.40E+02 |
| Tb11.02.4230 | IPR003590 |  | 590 | 617 | 2.10E+01 |
| Tb11.02.4230 | IPR003590 |  | 646 | 671 | 4.80E+01 |
| Tb11.02.4230 |  | RNI-like | 241 | 563 | 9.00E-31 |
| Tb11.02.4230 |  | RNI-like | 512 | 782 | 7.20E-25 |
| Tb927.1.4220 | IPR019024 | Ydr279p protein family (RNase H2 complex component) | 175 | 398 | 4.20E-15 |
| Tb927.10.12060 | IPR001810 | F-box domain | 82 | 129 | 1.90E-02 |
| Tb927.10.12060 | IPR001810 | F-box domain profile | 81 | 130 | 8.70E+00 |
| Tb927.10.12060 |  | F-box domain | 87 | 232 | 6.60E-06 |
| Tb927.10.9290 |  | P-loop containing nucleoside triphosphate hydrolases | 4 | 162 | 6.20E-12 |
| Tb927.3.1200 | IPR018249 | EF-hand calcium-binding domain profile | 634 | 669 | 8.80E+00 |
| Tb927.3.1200 |  | EF-hand | 635 | 691 | 1.60E-04 |
| Tb927.3.1230 | IPR000999 | RNase3 domain | 820 | 836 | 1.20E-05 |
| Tb927.3.1230 | IPR000999 | RNase III domain-like | 819 | 893 | 8.10E-07 |
| Tb927.3.1660 | IPR001357 | BRCA1 C Terminus (BRCT) domain | 485 | 522 | 3.10E-04 |
| Tb927.3.1660 | IPR001357 | BRCA1 C Terminus (BRCT) domain | 730 | 742 | 5.10E-01 |
| Tb927.3.1660 | IPR001357 | BRCT domain profile | 465 | 526 | 9.30E+00 |
| Tb927.3.1660 | IPR001357 | BRCT domain profile | 730 | 755 | 1.10E+01 |
| Tb927.3.1660 | IPR001357 | BRCT domain | 449 | 536 | 1.80E-10 |
| Tb927.3.1660 | IPR001357 | BRCT domain | 588 | 759 | 3.80E-12 |
| Tb927.3.2400 | IPR001594 | DHHC zinc finger domain | 121 | 176 | 2.20E-23 |
| Tb927.3.2400 | IPR001594 | Zinc finger DHHC-type profile | 121 | 171 | 2.60E+01 |
| Tb927.4.1110 |  | Metallo-dependent phosphatases | 20 | 331 | 8.60E-16 |
| Tb927.4.1970 | IPR002550 | Domain of unknown function DUF21 | 57 | 217 | 7.20E-30 |
| Tb927.4.1970 | IPR000644 | CBS domain pair | 270 | 379 | 2.10E-06 |
| Tb927.4.1970 | IPR000644 | CBS domain profile | 252 | 313 | 8.00E+00 |
| Tb927.4.1970 | IPR000644 | CBS domain profile | 320 | 386 | 8.20E+00 |
| Tb927.4.1970 |  | CBS-domain | 242 | 303 | 2.30E-06 |
| Tb927.4.1970 |  | CBS-domain | 310 | 396 | 9.90E-03 |
| Tb927.4.1970 | IPR018490 | cAMP-binding domain-like | 477 | 553 | 1.40E-04 |
| Tb927.4.5120 |  | Immunoglobulin | 174 | 1259 | 1.10E-02 |
| Tb927.4.570 | IPR016027 | Nucleic acid-binding proteins | 19 | 141 | 4.60E-04 |
| Tb927.4.570 | IPR016027 | Nucleic acid-binding proteins | 304 | 404 | 6.30E-05 |
| Tb927.4.570 | IPR016027 | Nucleic acid-binding proteins | 466 | 595 | 1.60E-11 |
| Tb927.5.1960 |  | dsRNA-binding domain-like | 351 | 378 | 1.80E-02 |
| Tb927.5.2970 | IPR011046 | WD40 repeat-like | 7 | 334 | 5.10E-13 |
| Tb927.5.3930 | IPR011701 | Major Facilitator Superfamily | 53 | 405 | 1.70E-17 |
| Tb927.5.3930 |  | Major facilitator superfamily (MFS) profile | 260 | 455 | 1.30E+01 |
| Tb927.5.3930 | IPR016196 | MFS general substrate transporter | 45 | 455 | 1.60E-31 |
| Tb927.7.360 | IPR008948 | L-aspartase-like | 161 | 289 | 1.60E-02 |
| Tb927.7.4630 |  | P-loop containing nucleoside triphosphate hydrolases | 4 | 187 | 5.90E-12 |
| Tb927.7.5060 | IPR003492 | CLN3 protein | 20 | 440 | 3.50E-12 |
| Tb927.7.5060 | IPR003492 | CLN3 Batten's disease protein (battenin) signature | 24 | 46 | 2.20E-20 |
| Tb927.7.5060 | IPR003492 | CLN3 Batten's disease protein (battenin) signature | 66 | 82 | 2.20E-20 |
| Tb927.7.5060 | IPR003492 | CLN3 Batten's disease protein (battenin) signature | 121 | 143 | 2.20E-20 |
| Tb927.7.5060 | IPR003492 | CLN3 Batten's disease protein (battenin) signature | 151 | 171 | 2.20E-20 |
| Tb927.7.5060 | IPR003492 | CLN3 Batten's disease protein (battenin) signature | 327 | 342 | 2.20E-20 |
| Tb927.7.5060 | IPR003492 | CLN3 Batten's disease protein (battenin) signature | 352 | 364 | 2.20E-20 |
| Tb927.7.5060 | IPR016196 | MFS general substrate transporter | 13 | 414 | 2.70E-05 |
| Tb927.7.5300 | IPR000008 | C2 domain | 12 | 92 | 5.20E-15 |
| Tb927.7.5300 | IPR002110 | Ankyrin repeat | 609 | 631 | 3.00E-05 |
| Tb927.7.5300 | IPR002110 | Ankyrin repeat | 648 | 668 | 4.60E-03 |
| Tb927.7.5300 | IPR020477 | C2 domain signature | 27 | 39 | 5.00E+00 |
| Tb927.7.5300 | IPR020477 | C2 domain signature | 51 | 64 | 5.00E+00 |
| Tb927.7.5300 |  | Proline rich extensin signature | 178 | 190 | 4.00E-10 |
| Tb927.7.5300 |  | Proline rich extensin signature | 192 | 208 | 4.00E-10 |
| Tb927.7.5300 |  | Proline rich extensin signature | 213 | 225 | 4.00E-10 |
| Tb927.7.5300 |  | Proline rich extensin signature | 231 | 252 | 4.00E-10 |
| Tb927.7.5300 |  | Proline rich extensin signature | 254 | 270 | 4.00E-10 |
| Tb927.7.5300 |  | Ankyrin repeat signature | 610 | 622 | 7.60E-02 |
| Tb927.7.5300 |  | Ankyrin repeat signature | 622 | 634 | 7.60E-02 |
| Tb927.7.5300 | IPR018029 | C2 domain profile | 11 | 92 | 1.10E+01 |
| Tb927.7.5300 | IPR002110 | Ankyrin repeat region circular profile | 609 | 708 | 1.30E+01 |
| Tb927.7.5300 | IPR002110 | Ankyrin repeat profile | 609 | 631 | 1.00E+01 |
| Tb927.7.5300 | IPR000008 |  | 11 | 107 | 6.90E-08 |
| Tb927.7.5300 | IPR002110 |  | 609 | 638 | 2.00E-01 |
| Tb927.7.5300 | IPR002110 |  | 648 | 677 | 4.80E+01 |
| Tb927.7.5300 | IPR008973 | C2 domain (Calcium/lipid-binding domain, CaLB) | 8 | 139 | 1.10E-15 |
| Tb927.7.5300 | IPR002110 | Ankyrin repeat | 551 | 706 | 1.60E-18 |
| Tb927.7.6370 | IPR018957 | Zinc finger, C3HC4 type (RING finger) | 16 | 53 | 6.20E-07 |
| Tb927.7.6370 | IPR001841 | Zinc finger RING-type profile | 16 | 54 | 1.20E+01 |
| Tb927.7.6370 | IPR001841 |  | 16 | 53 | 4.70E-04 |
| Tb927.7.6370 |  | RING/U-box | 6 | 83 | 1.90E-14 |
| Tb927.8.1250 | IPR006597 | Sel1 repeat | 82 | 120 | 6.90E-01 |
| Tb927.8.1250 | IPR006597 | Sel1 repeat | 121 | 156 | 8.40E-01 |
| Tb927.8.1250 | IPR006597 | Sel1 repeat | 157 | 190 | 7.60E-05 |
| Tb927.8.1250 | IPR006597 | Sel1 repeat | 241 | 272 | 3.10E-04 |
| Tb927.8.1250 | IPR006597 | Sel1 repeat | 273 | 313 | 1.10E+00 |
| Tb927.8.1250 | IPR006597 | Sel1 repeat | 314 | 361 | 1.10E-03 |
| Tb927.8.1250 | IPR006597 | Sel1 repeat | 362 | 397 | 4.40E-09 |
| Tb927.8.1250 | IPR006597 | Sel1 repeat | 581 | 618 | 1.80E-03 |
| Tb927.8.1250 | IPR006597 |  | 82 | 120 | 6.10E+00 |
| Tb927.8.1250 | IPR006597 |  | 121 | 156 | 1.20E-01 |
| Tb927.8.1250 | IPR006597 |  | 157 | 192 | 9.30E-05 |
| Tb927.8.1250 | IPR006597 |  | 236 | 272 | 4.30E-02 |
| Tb927.8.1250 | IPR006597 |  | 273 | 313 | 2.10E+00 |
| Tb927.8.1250 | IPR006597 |  | 314 | 361 | 4.40E+01 |
| Tb927.8.1250 | IPR006597 |  | 362 | 397 | 6.60E-06 |
| Tb927.8.1250 | IPR006597 |  | 398 | 429 | 3.40E+01 |
| Tb927.8.1250 | IPR006597 |  | 581 | 618 | 1.40E-02 |
| Tb927.8.1250 |  | HCP-like | 88 | 326 | 7.80E-24 |
| Tb927.8.1250 |  | HCP-like | 339 | 652 | 2.40E-20 |
| Tb927.8.8030 | IPR000348 | emp24/gp25L/p24 family/GOLD | 25 | 200 | 3.10E-19 |
