# Supplementary material for: Transcript Expression Analysis of Putative Trypanosoma brucei GPI-Anchored Surface Proteins during Development in the Tsetse and Mammalian Hosts
Source: PLoS Negl Trop Dis. 2012 Jun 19;6(6):e1708. doi: 10.1371/journal.pntd.0001708 (PMC3378594; doi:10.1371/journal.pntd.0001708)
Supplement: Table S6 — Bioinformatic analyses of the 81 putative genes that had a lower likelihood of encoding GPI-anchored proteins. These gene products either had TM domains, lacked signal peptide domains, or were predicted by only one of the GPI anchor prediction analysis. Forty-nine genes were conserved at the TriTryp level, having orthologs in the T. cruzi and L. major genomes. Twenty-one gene products had homologs identified in the available genome sequences of other species of Trypanosoma (T. cruzi, T. congolense, T. vivax, or T. b. gambiense) but not in L. major. Finally 12 genes examined were found to be present only in the genome of T. b. brucei, but not in any related kinetoplastid. (DOC) [file pntd.0001708.s006.doc]

| **Tb ORF** | **Kinetoplastid conservation^*^** | **GPI anchor prediction** | | **Signal Peptide with Cleavage Site^#^** | **Gly. Status^§^** | **TM^ø^** |
| --- | --- | --- | --- | --- | --- | --- |
|  |  | **BigPI** | **FragAnchor** |  |  |  |
| **Hypothetical Conserved** | |  |  |  |  |  |
| *Tb927.8.6570* | TriTryp | x | x | x | N-gly | 4 |
| *Tb09.211.2460* | TriTryp | x | x |  |  |  |
| *Tb927.7.4880* | TriTryp | x | x | x | N-gly | 1 |
| *Tb927.10.8930* | TriTryp | x |  |  |  |  |
| *Tb927.10.2820* | TriTryp | x |  |  |  |  |
| *Tb11.01.8300* | TriTryp | x | x |  |  |  |
| *Tb11.02.2220* | Tc, Tco | x |  | x |  | 5 |
| *Tb927.10.4010* | TriTryp | x |  |  |  |  |
| *Tb927.5.2970* | TriTryp | x | x |  |  |  |
| *Tb11.02.4230* | TriTryp | x | x |  |  |  |
| *Tb11.01.2940* | TriTryp | x |  |  |  |  |
| *Tb09.160.0430* | TriTryp | x |  | x | N-gly | 10 |
| *Tb927.4.3520* | TriTryp | x | x | x | N-gly | 4 |
| *Tb927.5.3930* | Tc | x | x |  |  | 12 |
| *Tb927.3.1200* | Tc, Tco, Tv | x | x |  |  |  |
| *Tb11.02.1920* | TriTryp | x |  |  |  |  |
| *Tb927.4.2940* | TriTryp | x |  |  | N- & O-gly |  |
| *Tb927.3.1660* | TriTryp | x |  | x |  |  |
| *Tb10.61.1700* | TriTryp | x | x |  | N-gly |  |
| *Tb927.1.4220* | Tc, Tco, Tv | x |  |  | N-gly |  |
| *Tb09.211.0820* | TriTryp | x | x |  | N-gly | 4 |
| *Tb09.211.3340* | TriTryp | x |  |  |  |  |
| *Tb927.10.7480* | TriTryp | x | x |  |  |  |
| *Tb927.6.1070* | TriTryp | x |  |  |  |  |
| *Tb09.211.4710* | TriTryp | x |  |  |  |  |
| *Tb927.7.5300* | TriTryp | x |  |  | N- & O-gly |  |
| *Tb927.5.1960* | TriTryp | x |  |  |  |  |
| *Tb927.7.4350* | TriTryp | x | x |  |  |  |
| *Tb927.5.3260* | TriTryp | x | x |  | N- & O-gly |  |
| *Tb927.10.12060* | Tc, Tco, Tv | x | x |  |  |  |
| *Tb927.7.3570* | TriTryp | x |  |  | N-gly |  |
| *Tb11.02.4850* | Tbg | x |  | x |  | 4 |
| *Tb11.01.6690* | TriTryp | x | x |  | N-gly | 6 |
| *Tb927.7.5710* | TriTryp | x | x | x | N-gly | 2 |
| *Tb927.1.530* | TriTryp | x |  |  | N-gly |  |
| *Tb09.211.4690* | TriTryp | x | x |  |  |  |
| *Tb11.02.2230* | TriTryp | x |  |  |  |  |
| *Tb927.4.570* | TriTryp | x |  |  |  |  |
| *Tb927.8.8030* | TriTryp | x | x | x | N-gly | 1 |
| *Tb927.7.2230* | TriTryp | x | x |  | N-gly | 1 |
| *Tb927.4.4550* | TriTryp | x |  |  |  |  |
| *Tb927.4.1970* | TriTryp | x |  |  | N-gly | 1 |
| *Tb927.4.3440* | TriTryp | x |  |  |  |  |
| *Tb927.8.5660* | TriTryp | x | x |  | N-gly | 3 |
| *Tb927.7.6370* | TriTryp | x |  |  |  |  |
| *Tb927.10.9290* | TriTryp | x |  | x | N-gly |  |
| *Tb11.01.4650* | TriTryp | x | x |  |  |  |
| *Tb927.7.4630* | TriTryp | x |  |  | N-gly |  |
| *Tb927.3.2780* | TriTryp | x |  |  |  |  |
| *Tb11.02.1540* | TriTryp | x |  |  | N- & O-gly |  |
| *Tb927.1.1500* | TriTryp | x |  |  | N- & O-gly |  |
| *Tb927.7.5060* | TriTryp | x |  |  | N-gly | 11 |
| *Tb927.4.5120* | TriTryp | x |  |  | N- & O-gly |  |
| *Tb927.10.11290* | TriTryp | x |  | x | N-gly |  |
| *Tb11.01.7980* | TriTryp | x | x |  |  |  |
| *Tb09.211.4070* | Tbg | x | x |  | N-gly |  |
| *Tb927.5.4020* | Tbg | x | x |  | N-gly |  |
| *Tb927.5.660* | Tbg | x | x |  | N-gly | 1 |
| *Tb06.3A7.960* | Tbg | x | x |  | N-gly |  |
| *Tb927.10.6470* | Tbg | x |  |  |  |  |
| *Tb927.3.2800* | Tbg, Tv | x | x |  |  |  |
| *Tb11.02.0390* | Tbg | x |  |  |  |  |
| *Tb927.5.4010* | Tbg | x | x |  | N- & O-gly |  |
| *Tb927.10.7170* | Tbg | x |  |  |  |  |
| *Tb11.02.2370* | Tco | x | x |  | N-gly |  |
| *Tb927.3.1230* | Tbg, Tco, Tv | x |  |  |  |  |
| *Tb09.244.2390* | Tbg | x | x |  | N-gly | 1 |
| *Tb927.3.3600* | Tbg | x |  |  |  | 5 |
| *Tb927.7.6330* | Tbg | x | x |  |  | 4 |
|  |  |  |  |  |  |  |
| **Hypothetical** |  |  |  |  |  |  |
| *Tb927.7.7310* |  | x | x | x | N-gly | 2 |
| *Tb11.02.2180* |  | x | x | x |  | 3 |
| *Tb11.02.1565* |  | x |  | x |  |  |
| *Tb927.10.3410* |  | x |  | x |  |  |
| *Tb927.8.7310* |  | x |  | x |  |  |
| *Tb927.8.7330* |  | x |  | x | N- & O-gly |  |
| *Tb927.1.5260* |  | x |  |  |  | 2 |
| *Tb927.8.3670* |  | x |  | x |  | 2 |
| *Tb09.v1.0970* |  | x | x |  |  | 2 |
| *Tb927.10.3620* |  | x |  | x |  |  |
| *Tb927.6.260* |  | x |  |  |  | 1 |
| *Tb927.8.490* |  | x |  | x | N-gly |  |

**^*^**TriTryp (*T. brucei*, *T. cruzi*, *L. major*); Tb = *T. brucei*; Tbg = *T. brucei* *gambiense*; Tco = *T. congolense*; Tc = *T. cruzi*; Tv = *T. vivax*

**^#^** signal peptide and signal sequence cleavage prediction made by publically available software

**^§^** N- or O-glycosylation status predicted by publically available software

**^ø^** number of transmembrane domains predicted by publically available software
