# Supplementary material for: Transcript Expression Analysis of Putative Trypanosoma brucei GPI-Anchored Surface Proteins during Development in the Tsetse and Mammalian Hosts
Source: PLoS Negl Trop Dis. 2012 Jun 19;6(6):e1708. doi: 10.1371/journal.pntd.0001708 (PMC3378594; doi:10.1371/journal.pntd.0001708)
Supplement: Table S7 — Developmental stage regulation and level of expression of gene products that were predicted to be less likely to contain GPI- anchor attachment signal domains. (DOC) [file pntd.0001708.s007.doc]

| **Tb ORF** | **SG°** | **PV°** | **MG°** | **BSF°** | **Expression Level^•^** |
| --- | --- | --- | --- | --- | --- |
| **SALIVARY GLAND SPECIFIC** | | | | | |
| *Tb927.10.3620* | 5 | 2 | 1 | 1 | High |
| *Tb11.01.2940* | 21 | 4 | 6 | 1 | Medium |
| *Tb927.10.2820* | 6 | 3 | 2 | 1 | Medium |
| *Tb927.4.2940* | 4 | 2 | 1 | 1 | Medium |
| *Tb927.3.1200** | 4 | 2 | nd | 1 | Medium |
| *Tb927.5.2970** | 23 | 10 | 1 | 10 | Medium |
| *Tb927.5.4010** | 5 | 1 | 1 | 2 | Medium |
| *Tb927.10.8930* | 4 | 1 | nd | 2 | Low |
| *Tb927.4.570* | 4 | 1 | nd | nd | Low |
|  |  |  |  |  |  |
| **SALIVARY GLAND and PROVENTRICULUS PREFERENTIAL** | | | | | |
| *Tb11.02.1920* | 8 | 7 | 1 | 1 | Medium |
| *Tb927.1.4220* | 4 | 5 | nd | 1 | Medium |
| *Tb09.211.4710* | 10 | 6 | 1 | 2 | Medium |
| *Tb927.5.1960* | 3 | 5 | 1 | 1 | Medium |
| *Tb927.4.4550* | 13 | 9 | nd | 1 | Medium |
|  |  |  |  |  |  |
| **SALIVARY GLAND and BSF PREFERENTIAL** | | | | | |
| *Tb927.8.490* | 1 | nd | nd | 2 | Low |
|  |  |  |  |  |  |
| **PROVENTRICULUS PREFERENTIAL** | | | | | |
| *Tb11.02.2370** | 1 | 26 | 6 | 1 | High |
| *Tb06.3A7.960** | 1 | 9 | 4 | 3 | Medium |
| *Tb11.02.4230** | 1 | 35 | 2 | 8 | Medium |
| *Tb927.3.2780* | 3 | 7 | nd | 1 | Medium |
| *Tb927.7.6370* | 2 | 5 | nd | 1 | Low |
| *Tb10.61.1700** | 1 | 3 | nd | 1 | Low |
| *Tb09.211.4690** | 1 | 4 | 1 | 1 | Low |
|  |  |  |  |  |  |
| **PROVENTRICULUS and BSF PREFERENTIAL** | | | | | |
| *Tb11.01.7980** | 1 | 21 | 3 | 16 | Medium |
| *Tb927.10.9290* | 1 | 5 | 2 | 4 | Medium |
| *Tb927.8.7330* | 1 | 2 | 1 | 3 | Medium |
|  |  |  |  |  |  |
| **MIDGUT and PROVENTRICULUS SPECIFIC** | | | | | |
| *Tb927.10.11290* | 2 | 11 | 10 | 1 | Medium |
|  |  |  |  |  |  |
| **MIDGUT SPECIFIC** | | | | | |
| *Tb927.3.1230* | 3 | 1 | 11 | 2 | High |
| *Tb927.5.4020** | 3 | 2 | 29 | 1 | Medium |
|  |  |  |  |  |  |
| **INSECT PREFERENTIAL** | |  |  |  |  |
| *Tb09.211.4070** | 6 | 5 | 11 | 1 | High |
| *Tb09.211.2460* | 2 | 2 | 2 | 1 | Medium |
| *Tb11.02.2230* | 1 | 2 | 2 | nd | Medium |
|  |  |  |  |  |  |
| **CONSTITUTITIVE** | | | | | |
| *Tb11.01.4650** | 1 | 2 | 1 | 1 | Medium |
| *Tb927.6.1070* | 1 | 2 | 1 | 2 | Low |
| *Tb927.10.7480* | 1 | 2 | 2 | 1 | Low |
| *Tb927.10.12060** | 2 | 3 | 2 | 1 | Low |
|  |  |  |  |  |  |
| **MISCELLANEOUS** | | | | | |
| *Tb927.6.440** | 1 | 5 | 4 | 7 | High |
| *Tb927.10.6470* | 1 | 2 | 6 | 6 | High |
| *Tb927.3.2800* | 5 | 1 | 5 | 8 | Medium |
| *Tb927.7.4350** | nd | 1 | 1 | 1 | Low |
| **Tb ORF** | **SG** | **PV** | **MG** | **BSF** | **Expression Level** |
| **SALIVARY GLAND SPECIFIC** | | | | | |
| *Tb927.10.3620* | 5 | 2 | 1 | 1 | High |
| *Tb11.01.2940* | 21 | 4 | 6 | 1 | Medium |
| *Tb927.10.2820* | 6 | 3 | 2 | 1 | Medium |
| *Tb927.4.2940* | 4 | 2 | 1 | 1 | Medium |
| *Tb927.3.1200** | 4 | 2 | nd | 1 | Medium |
| *Tb927.5.2970** | 23 | 10 | 1 | 10 | Medium |
| *Tb927.5.4010** | 5 | 1 | 1 | 2 | Medium |
| *Tb927.10.8930* | 4 | 1 | nd | 2 | Low |
| *Tb927.4.570* | 4 | 1 | nd | nd | Low |
|  |  |  |  |  |  |
| **SALIVARY GLAND and PROVENTRICULUS PREFERENTIAL** | | | | | |
| *Tb11.02.1920* | 8 | 7 | 1 | 1 | Medium |
| *Tb927.1.4220* | 4 | 5 | nd | 1 | Medium |
| *Tb09.211.4710* | 10 | 6 | 1 | 2 | Medium |
| *Tb927.5.1960* | 3 | 5 | 1 | 1 | Medium |
| *Tb927.4.4550* | 13 | 9 | nd | 1 | Medium |
|  |  |  |  |  |  |
| **SALIVARY GLAND and BSF PREFERENTIAL** | | | | | |
| *Tb927.8.490* | 1 | nd | nd | 2 | Low |
|  |  |  |  |  |  |
| **PROVENTRICULUS PREFERENTIAL** | | | | | |
| *Tb11.02.2370** | 1 | 26 | 6 | 1 | High |
| *Tb06.3A7.960** | 1 | 9 | 4 | 3 | Medium |
| *Tb11.02.4230** | 1 | 35 | 2 | 8 | Medium |
| *Tb927.3.2780* | 3 | 7 | nd | 1 | Medium |
| *Tb927.7.6370* | 2 | 5 | nd | 1 | Low |
| *Tb10.61.1700** | 1 | 3 | nd | 1 | Low |
| *Tb09.211.4690** | 1 | 4 | 1 | 1 | Low |
|  |  |  |  |  |  |
| **PROVENTRICULUS and BSF PREFERENTIAL** | | | | | |
| *Tb11.01.7980** | 1 | 21 | 3 | 16 | Medium |
| *Tb927.10.9290* | 1 | 5 | 2 | 4 | Medium |
| *Tb927.8.7330* | 1 | 2 | 1 | 3 | Medium |
|  |  |  |  |  |  |
| **MIDGUT and PROVENTRICULUS SPECIFIC** | | | | | |
| *Tb927.10.11290* | 2 | 11 | 10 | 1 | Medium |
|  |  |  |  |  |  |
| **MIDGUT SPECIFIC** | | | | | |
| *Tb927.3.1230* | 3 | 1 | 11 | 2 | High |
| *Tb927.5.4020** | 3 | 2 | 29 | 1 | Medium |
|  |  |  |  |  |  |
| **INSECT PREFERENTIAL** | |  |  |  |  |
| *Tb09.211.4070** | 6 | 5 | 11 | 1 | High |
| *Tb09.211.2460* | 2 | 2 | 2 | 1 | Medium |
| *Tb11.02.2230* | 1 | 2 | 2 | nd | Medium |
|  |  |  |  |  |  |
| **CONSTITUTITIVE** | | | | | |
| *Tb11.01.4650** | 1 | 2 | 1 | 1 | Medium |
| *Tb927.6.1070* | 1 | 2 | 1 | 2 | Low |
| *Tb927.10.7480* | 1 | 2 | 2 | 1 | Low |
| *Tb927.10.12060** | 2 | 3 | 2 | 1 | Low |
|  |  |  |  |  |  |
| **MISCELLANEOUS** | | | | | |
| *Tb927.10.6470* | 1 | 2 | 6 | 6 | High |
| *Tb927.3.2800* | 5 | 1 | 5 | 8 | Medium |
| *Tb927.7.4350** | nd | 1 | 1 | 1 | Low |

**°** SG = salivary gland, PV = proventriculus, MG = midgut, BSF= bloodstream form

**^•^** Expression level was categorized based on artificial numerical values

nd = not detected

& Normalized *in vivo* stage-specific gene expression profiles and levels for predicted proteins unlikely to be GPI-anchored based on (1) lack of correlation between BigPI and FragAnchor, or (2) absence of a predicted signal peptide sequence. Relative expression levels of experimental parasite genes were determined by calculating relative band intensities for each PCR product relative to trypanosome *alpha-tubulin* expression in the same sample. Fold change was calculated based on the value for the tissue with the lowest detectable expression for each gene. Transcripts were detected from 40 genes with lower likelihood of encoding GPI-anchored proteins. Nearly half were expressed by trypanosomes infecting the SG alone or the SG and another tissue (39.5%; 15/38). We found nearly one quarter of these genes to be specifically expressed by trypanosomes in the SG (23.7%; 9/38). The same number of trypanosome genes were expressed by parasites infecting either the PV alone or the PV and another tissue. In addition, 3 genes were preferentially expressed by trypanosomes inhabiting the MG, 1 of which was specifically upregulated in parasites infecting both the PV and MG organs. About 10% of genes encoding unlikely putative GPI-anchored proteins were expressed constitutively in all analyzed tissues. Approximately 7.7% were preferential to insect stages, as they were constitutive in the tsetse host, showing no tissue specificity, but were expressed at lower levels in the mammalian host. Most (23/38) of the genes were expressed at a moderate level or low level (10/38), leaving only 5 genes which had high levels of detectible transcripts.

*genes lacking predicted signal peptide sequence
